# Supplementary material for: Reporting quality of published reviews of commercial and publicly available mobile health apps (mHealth app reviews): a scoping review protocol
Source: BMJ Open. 2024 Jul 4;14(7):e083364. doi: 10.1136/bmjopen-2023-083364 (PMC11227806; doi:10.1136/bmjopen-2023-083364)
Supplement: Author's manuscript [file bmjopen-2023-083364.draft_revisions.pdf]

BMJ Open is committed to open peer review. As part of this commitment we make the peer review history of every article we publish publicly available.

When an article is published we post the peer reviewers' comments and the authors' responses online. We also post the versions of the paper that were used during peer review. These are the versions that the peer review comments apply to.

The versions of the paper that follow are the versions that were submitted during the peer review process. They are not the versions of record or the final published versions. They should not be cited or distributed as the published version of this manuscript.

BMJ Open is an open access journal and the full, final, typeset and author-corrected version of record of the manuscript is available on our site with no access controls, subscription charges or pay-per-view fees (http://bmjopen.bmj.com).

If you have any questions on BMJ Open's open peer review process please email info.bmjopen@bmj.com

BMJ Open

The reporting quality of published reviews of commercial and publicly available mobile health apps (mHealth app reviews): A scoping review protocol

Journal:	*BMJ Open*
Manuscript ID	bmjopen-2023-083364
Article Type:	Protocol
Date Submitted by the Author:	18-Dec-2023
Complete List of Authors:	Gasteiger, Norina; The University of Manchester, Division of Nursing, Midwifery and Social Work; NIHR Applied Research Collaboration Greater Manchester<br>Norman, Gill; The University of Manchester, Division of Nursing, Midwifery & Social Work; NIHR Applied Research Collaboration Greater Manchester<br>Grainger, Rebecca; University of Otago, Department of Medicine<br>Eost-Telling, Charlotte; The University of Manchester, School of Nursing, Midwifery & Social Work; NIHR Applied Research Collaboration Greater Manchester<br>Jones, Debra; The University of Manchester, School of Nursing, Midwifery & Social Work<br>Ali, Syed; The University of Manchester, Centre for Health Informatics, Division of Informatics, Imaging & Data Sciences, Manchester Academic Health Science Centre; NIHR Applied Research Collaboration Greater Manchester<br>van der Veer, Sabine N.; The University of Manchester, Centre for Health Informatics, Division of Informatics, Imaging & Data Sciences, Manchester Academic Health Science Centre; NIHR Applied Research Collaboration Greater Manchester<br>Ford, Claire; The University of Manchester, Division of Nursing, Midwifery & Social Work<br>Hall, Alex; The University of Manchester, Division of Nursing, Midwifery & Social Work<br>Law, Kate; The University of Manchester, Division of Nursing, Midwifery & Social Work; The Christie Hospital NHS Trust<br>Byerly, Matthew; The University of Kansas School of Medicine<br>Davies, Alan; The University of Manchester, Centre for Health Informatics, Division of Informatics, Imaging & Data Sciences, Manchester Academic Health Science Centre<br>Paripoorani, Deborah; Manchester Royal Infirmary, EMERGING Research Team; NIHR Applied Research Collaboration Greater Manchester<br>Shi, Chunhu; The University of Manchester, Division of Nursing, Midwifery & Social Work; NIHR Applied Research Collaboration Greater Manchester<br>Dowding, Dawn; The University of Manchester, Division of Nursing, Midwifery & Social Work; NIHR Applied Research Collaboration Greater Manchester

Keywords:	Information technology < BIOTECHNOLOGY & BIOINFORMATICS, Telemedicine < BIOTECHNOLOGY & BIOINFORMATICS, eHealth, STATISTICS & RESEARCH METHODS

SCHOLARONE™
Manuscripts

The reporting quality of published reviews of commercial and publicly available mobile health apps (mHealth app reviews): A scoping review protocol

Norina Gasteiger[1,2], Gill Norman[1,2,3], Rebecca Grainger[4], Charlotte Eost-Telling[1,2], Debra Jones[1], Syed Mustafa Ali[2,5], Sabine N. van der Veer[2,5], Claire R. Ford[1], Alex Hall[1], Kate Law[1,6], Matthew Byerly[7], Alan Davies[5], Deborah Paripoorani[2,8], Chunhu Shi[1,2] & Dawn Dowding[1,2]

1. Division of Nursing, Midwifery and Social Work, University of Manchester, Manchester, UK
2. National Institute for Health and Care Research Applied Research Collaboration Greater Manchester, Manchester, UK
3. NIHR Innovation Observatory, Population Health Sciences Institute, Newcastle University, Newcastle, UK
4. Department of Medicine, University of Otago Wellington, Wellington, New Zealand
5. Centre for Health Informatics, Division of Informatics, Imaging and Data Sciences, Manchester Academic Health Science Centre, University of Manchester, Manchester, UK
6. The Christie Hospital NHS Foundation Trust, Manchester, UK
7. The University of Kansas School of Medicine-Wichita, Wichita, Kansas, United States of America
8. EMERGING Research Team, Manchester Royal Infirmary, Manchester, UK

For peer review only - http://bmjopen.bmj.com/site/about/guidelines.xhtml

AUTHOR DETAILS

	ORCiD	Email address
Dr Norina Gasteiger	0000-0001-7801-7417	norina.gasteiger@manchester.ac.uk
Dr Gill Norman	0000-0002-3972-5733	gill.norman@manchester.ac.uk
Professor Rebecca Grainger	0000-0001-9201-8678	rebecca.grainger@otago.ac.nz
Dr Charlotte Eost-Telling	0000-0002-9568-3195	charlotte.eost-telling@manchester.ac.uk
Mrs Debra Jones	0000-0003-1479-3622	debra.jones@manchester.ac.uk
Mr Syed Mustafa Ali	0000-0001-9393-9049	syedmustafa.ali@manchester.ac.uk
Dr Sabine N. van der Veer	0000-0003-0929-436X	sabine.vanderveer@manchester.ac.uk
Dr Claire R. Ford	0000-0003-2950-1565	claire.ford@manchester.ac.uk
Dr Alex Hall	0000-0002-8849-744X	alex.hall@manchester.ac.uk
Ms Kate Law	0000-0002-0995-8309	kate.law@postgrad.manchester.ac.uk
Professor Matthew Byerly	0000-0001-7033-1268	mbyerly3@kumc.edu
Dr Alan Davies	0000-0001-5737-5629	alan.davies-2@manchester.ac.uk
Ms Deborah Paripoorani	N/A	Deborah.Paripoorani@mft.nhs.uk
Dr Chunhu Shi	0000-0003-0151-0451	chunhu.shi@manchester.ac.uk
Professor Dawn Dowding	0000-0001-5672-8605	dawn.dowding@manchester.ac.uk

For peer review only

ABSTRACT

Introduction

Reviews of commercial and publicly available smartphone (mobile) health applications (mHealth app reviews) are being undertaken and published in the peer-reviewed literature. However, there is variation in the conduct and reporting of mHealth app reviews, with no existing reporting guidelines. We aim to develop a PRISMA extension to guide the conduct and reporting of mHealth app reviews. This scoping review of published mHealth app reviews will explore their alignment, deviation, and modification to the PRISMA 2020 items for systematic reviews and identify a list of possible items to include in a PRISMA-App extension reporting guideline.

Method and analysis

We are following the Joanna Briggs Institute approach and Arksey and O'Malley's five-step process. Patient and public contributors, mHealth app review, digital health research and evidence synthesis experts, healthcare professionals and a specialist librarian gave feedback on the methods. We will search SCOPUS, CINAHL Plus, AMED, EMBASE, Medline, APA PsycINFO and ACM Digital Library for articles reporting mHealth app reviews and use a two-step screening process to identify eligible articles. Information on whether the authors have reported, or how they have modified the PRISMA 2020 items in their reporting will be extracted. Data extraction will also include the article characteristics, protocol and registration information, review question frameworks used, information about the search and screening process, how apps have been evaluated, and evidence of stakeholder engagement. This will be analysed using a content synthesis approach and presented using descriptive statistics and summaries.

Ethics and dissemination

Ethical approval is not required. The findings will be disseminated through peer-reviewed journal publications (shared on our project website and on the EQUATOR Network website where the PRISMA-App extension has been registered as under development), conference presentations and blog and social media posts in lay language.

Registration

This protocol is registered on OSF (osf.io/845fr).

For peer review only

KEYWORDS

app review; mHealth; smartphone; research methods; reporting; scoping review

STRENGTHS AND LIMITATIONS

- This review will be conducted systematically, with data extraction informed by the PRISMA 2020 reporting items and a previous scoping review.
- The protocol has had input from a multidisciplinary team of mHealth app review, digital health and evidence synthesis experts, healthcare professionals, a librarian, and patient and public contributors.
- The broad scope of health topics to be included in the review increases the generalisability of the findings.
- In line with scoping review guidance, a quality appraisal of the included studies will not be conducted.
- Only mHealth app reviews reported in English will be included, meaning some other relevant reviews published in other languages may not be included.

WORD COUNT: 2,719

For peer review only

INTRODUCTION

In 2021 it was recorded that there are more than 350,000 health applications (apps) (1). These applications are increasingly being integrated into healthcare; supporting professionals in their clinical practice (2) and empowering patients to manage and monitor their health conditions (3, 4). However, the quality and reliability of mobile health (mHealth) apps varies significantly (5), as developers can release smartphone health apps without any evaluation, meaning it is a challenge for health professionals and users without expertise in health research and digital technology to identify and evaluate the suitability of mHealth apps for their use.

This has led to the emergence of a new method: systematic reviews of commercial and publicly available mHealth apps (hereafter called mHealth app reviews). This provides a standard approach to identify mHealth apps relevant to a particular use case and assess aspects such as quality and functionality. Many mHealth app reviews have now been published, for varied topics including genetics (6), patient-reported outcomes in oncology (7), rheumatoid arthritis (8), strength training (9), menopause (10), exercise (11), hand hygiene (12), atrial fibrillation (13), pain (14) and smoking cessation (15). These reviews can serve as a valuable resource for healthcare decision-makers, practitioners, patients and the general public seeking high-quality mHealth apps; can identify gaps in the field and may guide researchers and industry in developing new products.

While mHealth app reviews share features with traditional systematic literature reviews, they differ substantially in their methods and reporting (16) due to the review being of commercial and publicly available products on app stores, instead of published literature. Examples of traditional systematic reviews of literature describing apps include those on monitoring and managing mental health symptoms (17) and self-managing pregnancy (18). While literature reviews can tell us about the effectiveness of apps which have been evaluated and the results published, they do not provide a comprehensive overview of all apps that are commercially or publicly available for use by patients, healthcare professionals and the public. mHealth app reviews also differ as there are no formal requirements for the protocol to be registered, searches take place on app libraries, screening often takes place on Excel (rather than using specifically designed tools like Rayyan (19) or Covidence (20)), and they are more challenging to replicate, as apps may emerge, disappear or be updated between searches (16).

The EQUATOR Network (21) provides an array of guidelines for reporting evaluations of digital technologies, such as the CONSORT-EHEALTH checklist (an extension of the CONSORT checklist tailored for reporting randomised controlled trials of web-based and mobile health interventions (22)), and guidance on reporting evaluations of specific technologies, such as sensors (23), mHealth interventions (24), telehealth in clinical trials (25) and smartphone-delivered ecological momentary assessments (26). There are also several extensions of the Preferred Reporting Items for Systematic Reviews and Meta-Analyses (PRISMA) reporting guidelines (27) available for different types of literature reviews.

In contrast, no reporting guidelines exist for mHealth app reviews, and we are not aware of any currently in development. The need for a reporting standard for health app-focused reviews was emphasised in a scoping review published in 2020 (28). The authors reviewed 26 mHealth app reviews published between 2012 and 2018 and found issues in their reporting. For example, the date of the searches was sometimes unclear (38%, 10/26) or absent (15%, 4/26). The number of reviewers involved was also poorly reported in 58% of reviews, and in 83% it was unclear whether screening

For peer review only - http://bmjopen.bmj.com/site/about/guidelines.xhtml

was performed independently. Another important finding was the absence of clinical recommendations or reporting on clinical efficacy, found in 77% of the included reviews.

If a key purpose of an mHealth app review is for people to be able to identify the best product for a particular purpose, it is important to further explore how app review authors have evaluated the apps and reported on their outcomes. For example, some previous app reviews have considered the accessibility of the apps by generating readability metrics on the written content (10, 29, 30). This is especially important for patient or public-facing apps. There are also various approaches to reporting on efficacy (i.e., that the app results in intended outcomes). This may include searching for evidence of a previous evaluation within the app itself, on developers' websites, or for published literature or conference abstracts in academic databases. For example the Mobile App Rating Scale (MARS) (31) is commonly used in mHealth app reviews and has an item which addresses *whether an app has been trialled or tested.* Previous examples of app reviews have approached this item by excluding the item entirely (8) or searching Google Scholar (6, 7, 10, 12, 14). It is unclear how other authors have evaluated the apps and how this has informed their recommendations of apps as being high-quality. Understanding the nature of the evaluations (especially efficacy evidence) seems essential in mHealth app reviews as readers, including health and care workers, patients and the public, and healthcare decision-makers may use these to choose which apps to use.

We have previously discussed the methodological considerations for conducting systematic mHealth app reviews; introducing a 7-step method and the TECH framework (Target user, Evaluation focus, Connectedness and the Health domain) for developing research (review) questions and determining app eligibility criteria (16). This is the first stage of a broader project that aims to systematise the process of conducting and reporting mHealth app reviews.

The next step is to develop reporting guidelines to support authors of mHealth app reviews in transparently presenting their methods and findings. The field of app reviews is rapidly developing and expanding, so a new scoping review is required to update that previously reported, which had a search date of 2018 (28). A preliminary search of the Cochrane Database of Systematic Reviews, Google Scholar and JBI Evidence Synthesis was conducted, and no current or ongoing systematic reviews or scoping reviews on the topic were identified. Therefore, we are undertaking a scoping review to build on and update the scoping review by Grainger et al. (28).

OBJECTIVES

In line with guidance for developing reporting guidelines (32), the next step is to search for relevant evidence on the quality of the reporting of published mHealth app reviews. The aim of this work is, therefore, to conduct a scoping review on published mHealth app reviews to explore their alignment, deviation and modification to the PRISMA 2020 items and identify a list of possible items to include in a new PRISMA-App review reporting guideline extension.

METHODS

Scoping review

The methods for this scoping review were developed in alignment with the Joanna Briggs Institute approach for scoping reviews (33) and reviewed by a group of patient and public contributors and an advisory group consisting of mHealth app review, digital health research and evidence synthesis

For peer review only

experts and NHS healthcare professionals interested in app reviews. The review will be carried out using the five-step process for conducting scoping reviews, originally outlined by Arksey and O'Malley (34). This protocol has already been registered and made publicly available on OSF (https://osf.io/). The review will be reported using the PRISMA-ScR extension guidelines for scoping reviews (35).

Procedures

Identifying the initial research question

We used the Study, Data, Methods and Outcomes (SDMO) acronym to inform our research questions and eligibility criteria, which has been recommended when conducting reviews on methodology or theory (36). Additionally, as suggested by Levac et al. (37), we considered the purpose and expected outputs of the review to assist in writing the research questions. The purpose and expected outputs are primarily a list of potential reporting items used to inform the future PRISMA-App review guideline. Focussing on the PRISMA 2020 (27) items is appropriate as many app review authors already informally use the PRISMA items to report their work or have attempted to amend the PRISMA flowchart when reporting their app search and screening process (6-10, 12-15).

The second question seeks to understand what outcomes were evaluated in mHealth app reviews (e.g., usability, functionality, privacy, accessibility and efficacy). This builds on the previous review (28), which found that most of the app reviews did not make clinical recommendations or report clinical efficacy (i.e., whether the app could meet desired outcomes in a clinical context). We are therefore interested in understanding what the outcomes were in general, and whether any of the app reviews reported on efficacy in any other sense such as satisfaction, increased knowledge or perceived support.

The two key questions are:

1. In published reviews of commercial and publicly available mHealth apps how does reporting align with or deviate from the PRISMA 2020 items? Have authors used items that directly align with PRISMA 2020 items or have these been modified?
2. What outcomes did the mHealth app reviews evaluate and report on?

Identifying relevant studies

We will search the SCOPUS, CINAHL Plus (via EBSCO), AMED (via Ovid), EMBASE (via Ovid), Medline (via Ovid), APA PsycINFO and ACM Digital Library databases for published mHealth app reviews, under the guidance of a teaching and learning librarian who has given input on the search strategy. Reference lists of eligible articles will also be hand-searched for additional sources (snowballing), while a forward citation approach will be used to identify app reviews that have cited earlier published work.

The key terms used to build the search strategy are shown in Table 1, with examples from AMED, EMBASE, Medline and APA PsycINFO presented in Appendix 1. Where appropriate, subject headings will be applied in the databases. The keywords will be separated by the 'OR' Boolean operator. The technology and review type keywords will be separated by the 'AND' Boolean operator. The proximity function using five words will be used for the review type keywords, to

For peer review only - http://bmjopen.bmj.com/site/about/guidelines.xhtml

include different variations of app review, such as review of apps, review of smartphone apps, or review of patient-facing mobile health applications.

Publication date will be limited from 1st January 2007, as the first iPhone (and first smartphone) was introduced on 29th June 2007, so there will be no apps available to review before 2007.

Table 1. Keywords used to identify literature.

Technology	Review type
• Mobile<br>• Smartphone<br>• "Cell phone"<br>• mHealth<br>• "mobile health"<br>• eHealth<br>• Tele* (to include telehealth, telemedicine and other variations)	(App or apps or application or applications) adj5[1] review

Study selection

Table 2 presents the eligibility criteria for the literature, using the SDMO acronym (36). Broadly, our inclusion criteria are:

- Reviews of commercial and publicly available mobile (smartphone) apps published in English and after 1 January 2007 (types of study)
- that have a health focus (type of data),
- include any method and measure of evaluating apps (e.g., MARS or user ratings) and,
- have any outcome or focus, including focussing on the availability of apps or evaluation (e.g., quality, functionality, privacy and security or adherence to clinical guidelines).

We will only include mHealth app reviews published in English. However, we will separately list articles that were excluded due to language, which can enable others to easily identify these papers for subsequent reviews.

[1] The exact wording depends on the database and may include adj5, W/5 or N5. The ACM Digital Library does not allow the proximity function, so "app* review" will be used instead.

For peer review only - http://bmjopen.bmj.com/site/about/guidelines.xhtml

Table 2. Eligibility criteria for the mHealth app reviews to be included in the scoping review.

SDMO Acronym	Inclusion criteria	Exclusion criteria	No limits
Types of study	Reviews of commercial and publicly available mobile apps<br>• *Must be focussed on smartphone (mobile) apps.*<br>• *Can be identified as systematic, scoping or without a specific approach named.*<br>• *Some app reviews may be combined with other literature reviews or reviews of other apps. These will only be included if detail is reported separately on the smartphone app review methods and results.*<br>• *Reviews including other technology (e.g., iPads, digital assistants, virtual reality headsets or smartwatches) will only be included if the focus is on smartphone apps and the other technology is used only to operationalise some of the functions.*<br><br>English language<br><br>Published on or after 1 January, 2007	Literature reviews<br><br>Reviews of other technology or apps (e.g., websites, computer apps, iPad apps)<br><br>Full-text not available<br>• *Exclude abstracts and documents where there is insufficient information or the full-text is not available.*<br><br>Not in English<br><br>Published before 1 January, 2007	Document type<br>• *Any document type will be included, if there is a full-text available so that enough information can be extracted (e.g., full-length conference papers, journal articles, book chapters).*<br><br>Smartphone device<br><br>Operating system requirements<br><br>App markets<br><br>Geography (location)
Types of data	Health focus<br><br>*Must be focussed on a health topic, whereby the apps are marketed for physical or mental health or general well-being. This may include (but is not limited to) apps that educate, empower or inform users on a health topic (e.g., genetics), self-monitor/manage or change health behaviours (e.g., sleep, nutrition, exercise or smoking cessation) or are used for social support or in health systems or by patients, administrators and health and care workers or decision-makers (e.g., screening, diagnosis, triage, appointment-booking, remote monitoring, decision-making, training and treatment).*	Not focussed on health topics	Health topic<br>• *Apps can be for any health topic.*<br><br>Intended users of apps<br>• *Apps can be for any stakeholder, including patients, the public, health professionals and the health system.*
Types of methods	Any method (and measure) of evaluating apps can be included, such as using validated measures (e.g., MARS (31)), synthesising content presented within the app or user ratings and reviews on app markets.	N/A	App evaluation measures and methods.
Types of outcomes	Any outcome, including those related to evaluating app quality, functionality, privacy and security, accessibility or efficacy. This would also include app reviews that simply focus on identifying which apps were available, summarised their content or described the extent to which they adhere to best clinical practice/guidelines.	N/A	Any outcomes.

For peer review only

For peer review only - http://bmjopen.bmj.com/site/about/guidelines.xhtml

The final search results will be imported into Rayyan (19) for deduplication and screening. As suggested by Levac et al. (37), the screening process will be iterative and use a team-based approach. An initial meeting will be held with all researchers involved in the screening process to discuss interpretation of the eligibility criteria and reach shared understanding after an initial set of records have been pilot screened. A two-step process will then be followed. First, two researchers will independently review each abstract/title against the eligibility criteria. Second, the full text of records potentially eligible based on abstract/title screening will be reviewed by two researchers independently. A meeting will be held after the second stage to discuss and reach consensus where there is disagreement. A third reviewer will make the final decision if consensus cannot be reached. Depending on the number of articles, two teams of two researchers may perform the screening process, with a fifth researcher available to resolve disagreements. The search and screening process will be presented as a flowchart.

Charting the data

A data extraction sheet will be created in Excel using headers related to our 7-step method and TECH framework (16), the PRISMA 2020 items (27), whether and how authors modified them, additional information reported, and methods used to appraise the apps' quality, functionality or efficacy. Some items have also been taken from the review conducted by Grainger et al. (28) as these capture details unique to app reviews (e.g., how app stores were searched). Table 3 presents the data extraction items.

Table 3. Information to be extracted from the articles.

**Article characteristics**	
Title	• Title of the article.<br>• Does it name a review method in the title (e.g., systematic, scoping, app review)?
Date	• Year of publication.
Journal	• Name of the journal the app review is published in.<br>• Name and contact information of the editor-in-chief.[2]
Authors	• Name and contact information of all authors.[2]<br>• Source of funding (if any).
Objective	• Aim or research question.
Topic and context	• The health problem being explored.<br>• App target user.<br>• Context: where the app is to be used, including location of care (acute, primary health care, community, long-term care, etc.) and geographical location.
**Protocol and registration**	
A priori review registration	• Was the study protocol registered and was the protocol available?[3]
**Review question**	
Review question frameworks	• Was a framework used to write the review question (e.g., PICO)? If so, which?<br>• Did this align with any of the TECH components? If so, which?
**Reporting guidelines**	
Reporting guidelines	• Did authors state which reporting guideline they used? If so, which?

[2] This information will not be published publicly as part of the review. It will be used to create a database of authors of mHealth app reviews and journals who have published them. This is required for the next step of the project- the Delphi (consensus-building) study, as mHealth experts will be participants and potential partners in this process.
[3] These data extraction items were taken from the scoping review conducted by Grainger et al. (28).

For peer review only

	• Did the authors clearly mention amending any guidelines?
Alignment, deviation and modification to the PRISMA (2020) items (see Appendix 2)	• For each of the PRISMA (2020) items, identify whether the information was reported as is, or how this was modified (if applicable).
**Search and screening**	
Flow charts/diagrams	• Did the authors present a flow diagram for the search and screening process?<br>• Did the authors report a PRISMA (2020) flow chart for new or updated systematic reviews (see Appendix 3 and 4)?<br>• Were amendments made to the PRISMA (2020) flow charts (see Appendix 3 and 4)? If yes, which?
App store search	• Were the apps store(s) searched described including the a) keywords, b) countries/location and c) a clear description of dates of the search?[3]<br>• Was the method for identifying and removing duplicate apps clearly described, including metadata (e.g., version numbers) used to determine if apps were duplicates?[3]<br>• Were the number and independence of people screening apps described?[3]<br>• Were limits on inclusion of apps based on other factors clearly described (lite or full version, paid or free versions, non-English)?[3]<br>• Where the same app featured in different app stores (multiplatform apps), was there a clear statement of which apps were included?[3]<br>• When apps were downloaded to phones for data extraction, were the phone model and version of the operating system clearly reported?[3]
**Evaluating the apps and making recommendations**	
Outcomes	• Which outcomes did the review evaluate? e.g., quality, functionality, usability, privacy, efficacy, accessibility or other
Quality, functionality, usability and other assessments	• Was a best practice content tool (e.g., clinical guideline) used to evaluate app content or quality? Was the source identified, were any modifications made described, and was the use of the guideline justified?[3]<br>• Was an app quality, functionality and/or usability evaluation undertaken using established measures and frameworks (e.g., MARS)?<br>• Are any other tools/instruments used to evaluate app quality and/or usability, and was the source of these described?[3]<br>• Were security and privacy considered? If so, what aspects were included? e.g., login, password, privacy policy, access to microphone or camera, encryption or data sharing.
Efficacy	• Did the authors report on the efficacy of the apps?<br>• If yes, where did they obtain this information and what methods were used?
Accessibility	• Was the accessibility of the apps evaluated, considering different needs of target users?
Recommendations	• Were any apps recommended for use in the clinical setting or by people with chronic health conditions?[3]<br>• Were any apps recommended overall? What informed this recommendation?
**Stakeholder engagement or consultation**	
Patient, public and expert engagement	• Were any patients or members of the public involved in the app review?<br>• Were any other stakeholders involved or consulted (e.g., industry partners, clinicians or software developers)? If so, which?<br>• Was a lay summary provided? If so, were any apps clearly recommended?
**Other**	

For peer review only - http://bmjopen.bmj.com/site/about/guidelines.xhtml

Other domains	• Any other domains reported on? If yes, what and how?

Consistent with recommendations by Levac et al. (37), we will take an iterative approach to charting by continually updating the data-charting form as needed. The research team will first pilot the data extraction sheet, by extracting the data from one app review, with discussion afterward to ensure consistency in interpretation of the items. Data will then be extracted from the other articles, with one author extracting the information and another checking this. Depending on the final number of included articles, this will be split between the researchers.

It has been suggested that some scoping reviews should also include quality assessments of the articles (37). However, as this is not the focus of our review and as no specific quality assessment tool currently exists for mHealth app reviews, the quality of the included studies will not be assessed.

Collating, summarising, and reporting the results

Similar to the previous review (28) and as recommended by Arksey and O'Malley (34), we will report data as frequencies (where possible) to determine which items were reported as is, or whether they were modified.

Information that cannot be reported as frequencies, on how the PRISMA 2020 items were modified and how other relevant information was reported will be summarised using a content synthesis approach, to help inform amendments to the existing guideline and to identify new items.

The results overall will be reported using descriptions and examples, while some of the numerical results will also be presented using tables and figures.

Strengths and limitations

This scoping review will be conducted in a systematic and rigorous manner, with data extraction informed by the existing PRISMA 2020 (27) reporting items and a previous scoping review (28). It also adheres to existing guidance on conducting scoping reviews, including from the Joanna Briggs Institute (33) and Arksey and O'Malley (34), and has had input from a multidisciplinary team of mHealth app review, digital health and evidence synthesis experts, NHS healthcare professionals, a librarian and patient and public contributors. The breadth of its scope of health topics (and methodological focus), also means that the findings will be widely generalisable.

A fundamental limitation is the inability to assess the quality of the included reviews, due to an absence of quality appraisal tools for reviews of commercial and publicly available mHealth apps. This limitation means that low quality app reviews may also contribute to the development of the future PRISMA-App reporting guideline. However, we will mitigate this in the next phase of the project, in which a Delphi study with experts will help to prioritise the items. Another limitation concerns restricting the included mHealth app reviews to those reported in English which may lead to other relevant reviews being excluded.

Patient and public involvement

We have established a PPIE group to give feedback on all aspects of our project. The group has been consulted to provide input on the protocol and suggested additional items that should be extracted

For peer review only

(i.e., whether the accessibility of the apps was evaluated and whether a lay summary was provided). They also gave ideas for how the findings should be disseminated. We will continue to consult with them throughout the review; this will inform the iterative aspects of the scoping review process and will help to guide the findings and their dissemination.

ETHICS AND DISSEMINATION

Ethical approval is not required to conduct this scoping review which will use only previously published data.

The findings of this scoping review will be disseminated through peer-reviewed journal publications which will be shared on our project website and on the EQUATOR Network website where the PRISMA-App review extension has been registered as under development, in addition to conference presentations and blog posts and short summaries in lay language on professional social media.

CONCLUSION

This protocol describes how we will conduct a scoping review on published mHealth app reviews to explore their alignment, deviation and modification to the PRISMA 2020 items and identify a list of possible items to include in the new PRISMA-App review reporting guideline extension. The results will inform the next phase in developing the new PRISMA-App review reporting guideline: a Delphi study to reach a consensus on which items are most relevant and important to include in the guideline.

DECLARATIONS

Author contributions

NG, GN and DD designed the study. NG wrote the first draft of the manuscript, with support from GN. All authors revised and approved the final manuscript.

Funding

This work is funded by the National Institute for Health and Care Research Applied Research Collaboration Greater Manchester (Grant award: NIHR200174). The views expressed in this publication are those of the authors and not necessarily those of the National Institute for Health and Care Research or the Department of Health and Social Care.

Acknowledgements

We would like to acknowledge Mr Michael Stevenson for assisting with the search strategy. We would also like to thank our patient and public contributors for their input- thank you Amber McAvoy, Eric Lowndes, Ashgan Mahyoub and Beatrice Namu. We acknowledge Mrs Amy Vercell and Dr Lisa McGarrigle who are part of our team.

Competing interests

The authors have no competing interests to declare.

For peer review only - http://bmjopen.bmj.com/site/about/guidelines.xhtml

Patient consent for publication

Not required.

Data availability

No new data has been generated for this protocol.

References

1. IQVIA. Digital Health Trends 2021: Innovation, evidence, regulation and adoption. [Internet]. 2021. Available from: https://www.iqvia.com/insights/the-iqvia-institute/reports-and-publications/reports/digital-health-trends-2021
2. Mayer MA, Rodríguez Blanco O and Torrejon A. Use of Health Apps by Nurses for Professional Purposes: Web-Based Survey Study. *JMIR Mhealth Uhealth.* 2019;7(11):e15195. 2019/11/05. doi: 10.2196/15195.
3. Dsouza SM, Shetty S, Venne J, et al. Effectiveness of self-management applications in improving clinical health outcomes and adherence among diabetic individuals in low and middle-income countries: a systematic review. *BMJ Open.* 2022;12(11):e060108. 2022/11/16. doi: 10.1136/bmjopen-2021-060108.
4. Doumen M, De Cock D, Van Lierde C, et al. Engagement and attrition with eHealth tools for remote monitoring in chronic arthritis: a systematic review and meta-analysis. *RMD Open.* 2022;8(2) 2022/10/28. doi: 10.1136/rmdopen-2022-002625.
5. Larsen ME, Huckvale K, Nicholas J, et al. Using science to sell apps: Evaluation of mental health app store quality claims. *NPJ Digit Med.* 2019;2:18. 2019/07/16. doi: 10.1038/s41746-019-0093-1.
6. Gasteiger N, Vercell A, Davies A, et al. Patient-facing genetic and genomic mobile apps in the UK: a systematic review of content, functionality, and quality. *J Community Genet.* 2022;13(2):171-182. 2022/02/20. doi: 10.1007/s12687-022-00579-y.
7. Vercell A, Gasteiger N, Yorke J, et al. Patient-facing cancer mobile apps that enable patient reported outcome data to be collected: A systematic review of content, functionality, quality, and ability to integrate with electronic health records. *Int J Med Inform.* 2022;170:104931. 2022/12/04. doi: 10.1016/j.ijmedinf.2022.104931.
8. Grainger R, Townsley H, White B, et al. Apps for People With Rheumatoid Arthritis to Monitor Their Disease Activity: A Review of Apps for Best Practice and Quality. *JMIR Mhealth Uhealth.* 2017;5(2):e7. doi: 10.2196/mhealth.6956.
9. McGarrigle L, Boulton E and Todd C. Map the apps: a rapid review of digital approaches to support the engagement of older adults in strength and balance exercises. *BMC Geriatr.* 2020;20(1):483. 2020/11/20. doi: 10.1186/s12877-020-01880-6.
10. Paripoorani D, Gasteiger N, Hawley-Hague H, et al. A systematic review of menopause apps with an emphasis on osteoporosis. *BMC Womens Health.* 2023;23(1):518. 2023/09/29. doi: 10.1186/s12905-023-02612-9.
11. Knight E, Stuckey MI, Prapavessis H, et al. Public Health Guidelines for Physical Activity: Is There an App for That? A Review of Android and Apple App Stores. *JMIR mHealth uHealth.* 2015;3(2):e43. doi: 10.2196/mhealth.4003.
12. Gasteiger N, Dowding D, Ali SM, et al. Sticky apps, not sticky hands: A systematic review and content synthesis of hand hygiene mobile apps. *J Am Med Inform Assoc.* 2021;28(9):2027-2038. 2021/06/29. doi: 10.1093/jamia/ocab094.
13. Pearsons A, Hanson CL, Gallagher R, et al. Atrial fibrillation self-management: a mobile telephone app scoping review and content analysis. *Eur J Cardiovasc Nurs.* 2021;20(4):305-314. 2021/02/24. doi: 10.1093/eurjcn/zvaa014.
14. Ali SM, Lau WJ, McBeth J, et al. Digital manikins to self-report pain on a smartphone: A systematic review of mobile apps. *Eur J Pain.* 2021;25(2):327-338. 2020/10/29. doi: 10.1002/ejp.1688.

For peer review only - http://bmjopen.bmj.com/site/about/guidelines.xhtml

15. Seo S, Cho SI, Yoon W, et al. Classification of Smoking Cessation Apps: Quality Review and Content Analysis. *JMIR Mhealth Uhealth.* 2022;10(2):e17268. 2022/02/18. doi: 10.2196/17268.
16. Gasteiger N, Dowding D, Norman G, et al. Conducting a systematic review and evaluation of commercially available mobile applications (apps) on a health-related topic: the TECH approach and a step-by-step methodological guide. *BMJ Open.* 2023;13(e073283). doi: 10.1136/bmjopen-2023-073283.
17. Wang K, Varma DS and Prosperi M. A systematic review of the effectiveness of mobile apps for monitoring and management of mental health symptoms or disorders. *Journal of Psychiatric Research.* 2018;107:73-78. doi: https://doi.org/10.1016/j.jpsychires.2018.10.006.
18. Iyawa GE, Dansharif AR and Khan A. Mobile apps for self-management in pregnancy: a systematic review. *Health and Technology.* 2021;11(2):283-294. doi: 10.1007/s12553-021-00523-z.
19. Ouzzani M, Hammady H, Fedorowicz Z, et al. Rayyan—a web and mobile app for systematic reviews. *Syst Rev.* 2016;5(1):210. doi: 10.1186/s13643-016-0384-4.
20. Veritas Health Innovation. Covidence systematic review software. [Internet]. Available from: www.covidence.org
21. Enhancing the QUAlity and Transparency Of health Research Network. Your one-stop-shop for writing and publishing high-impact health research. [Internet]. 2023. Available from: https://www.equator-network.org/
22. Eysenbach G. CONSORT-EHEALTH: improving and standardizing evaluation reports of Web-based and mobile health interventions. *J Med Internet Res.* 2011;13(4):e126. 2012/01/03. doi: 10.2196/jmir.1923.
23. Manta C, Mahadevan N, Bakker J, et al. EVIDENCE Publication Checklist for Studies Evaluating Connected Sensor Technologies: Explanation and Elaboration. *Digit Biomark.* 2021;5(2):127-147. 2021/06/29. doi: 10.1159/000515835.
24. Agarwal S, LeFevre AE, Lee J, et al. Guidelines for reporting of health interventions using mobile phones: mobile health (mHealth) evidence reporting and assessment (mERA) checklist. *Bmj.* 2016;352:i1174. 2016/03/19. doi: 10.1136/bmj.i1174.
25. Rhon DI, Fritz JM, Kerns RD, et al. TIDieR-telehealth: precision in reporting of telehealth interventions used in clinical trials - unique considerations for the Template for the Intervention Description and Replication (TIDieR) checklist. *BMC Medical Research Methodology.* 2022;22(1):161. doi: 10.1186/s12874-022-01640-7.
26. Dao KP, De Cocker K, Tong HL, et al. Smartphone-Delivered Ecological Momentary Interventions Based on Ecological Momentary Assessments to Promote Health Behaviors: Systematic Review and Adapted Checklist for Reporting Ecological Momentary Assessment and Intervention Studies. *JMIR Mhealth Uhealth.* 2021;9(11):e22890. 2021/11/23. doi: 10.2196/22890.
27. Page MJ, McKenzie JE, Bossuyt PM, et al. The PRISMA 2020 statement: an updated guideline for reporting systematic reviews. *BMJ.* 2021;372:n71. doi: 10.1136/bmj.n71.
28. Grainger R, Devan H, Sangelaji B, et al. Issues in reporting of systematic review methods in health app-focused reviews: A scoping review. *Health Informatics Journal.* 2020;26(4):2930-2945. doi: 10.1177/1460458220952917.
29. Gasteiger N, Vercell A, Davies A, et al. Patient-facing genetic and genomic mobile apps in the UK: a systematic review of content, functionality, and quality. *Journal of Community Genetics.* 2022;13(2):171-182. doi: 10.1007/s12687-022-00579-y.
30. Vercell A, Gasteiger N, Yorke J, et al. Patient-facing cancer mobile apps that enable patient reported outcome data to be collected: A systematic review of content, functionality, quality, and ability to integrate with electronic health records. *Int J Med Inform.* 2023;170:104931. 2022/12/04. doi: 10.1016/j.ijmedinf.2022.104931.
31. Stoyanov S, Hides L, Kavanagh D, et al. Mobile app rating scale: a new tool for assessing the quality of health mobile apps. *JMIR mHealth uHealth.* 2015;3(1):e27. doi: 10.2196/mhealth.3422.
32. Moher D, Schulz KF, Simera I, et al. Guidance for Developers of Health Research Reporting Guidelines. *PLOS Medicine.* 2010;7(2):e1000217. doi: 10.1371/journal.pmed.1000217.

For peer review only - http://bmjopen.bmj.com/site/about/guidelines.xhtml

33. JBI Scoping Review Methodology Group. JBI manual for evidence synthesis: scoping reviews chapter. [Internet]. 2023. Available from: https://jbi.global/scoping-review-network/about

34. Arksey H and O'Malley L. Scoping studies: Towards a methodological framework. *International Journal of Social Research Methodology* 2005;8(1):19-32. doi: 10.1080/1364557032000119616.

35. Tricco A, Lillie E, Zarin W, et al. PRISMA extension for scoping reviews (PRISMA-ScR): checklist and explanation. *Ann Intern Med.* 2018;169(7):467–473. doi: 10.7326/M18-0850.

36. Clarke M, Oxman A, Paulsen E, et al. Appendix A: Guide to the contents of a Cochrane Methodology protocol and review. *Cochrane Handbook for Systematic Reviews of Interventions Version 510*. The Cochrane Collaboration, 2011.

37. Levac D, Colquhoun H and O'Brien KK. Scoping studies: Advancing the methodology. *Implement Sci.* 2010;5(69):1-9. doi: 10.1186/1748-5908-5-69.

For peer review only

Appendix 1. Examples of the searches conducted on four databases.

	**AMED (Allied and Complementary Medicine) <1985 to October 2023>**	**Embase <1980 to 2023 Week 45>**	**Ovid MEDLINE(R) <1946 to October Week 5 2023>**	**APA PsycInfo <1806 to November Week 1 2023>**
**Technology**	Mobile.mp	mobile phone/	Mobile.mp.	Mobile Phones/
	Smartphone.mp	smartphone/	Smartphone/	Mobile Health/
	mHealth.mp	mobile health application/	Cell Phone/	eHealth.mp
	"mobile health".mp	telehealth/	mHealth.mp	telemedicine/
	"Cell phone".mp		"mobile health".mp	
	eHealth.mp		eHealth.mp	
	Tele*.mp		Telemedicine/ or Tele*.mp	
**Review type**	((App or apps or application or applications) adj5 review).mp	((app or apps or application or applications) adj5 review).mp	((App or apps or application or applications) adj5 review).mp	((App or apps or application or applications) adj5 review).mp
**Limits**	limit to (english and yr="2007 -Current")	limit to (english language and yr="2007 -Current")	limit to (english language and yr="2007 -Current")	limit to (english language and yr="2007 -Current")

For peer review only

Appendix 2. PRISMA (2020) checklist.

From: Page MJ, McKenzie JE, Bossuyt PM, Boutron I, Hoffmann TC, Mulrow CD, et al. The PRISMA 2020 statement: an updated guideline for reporting systematic reviews. BMJ 2021;372:n71. doi: 10.1136/bmj.n71

Section and Topic	Item #	Checklist item	Location where item is reported
**TITLE**			
Title	1	Identify the report as a systematic review.	
**ABSTRACT**			
Abstract	2	See the PRISMA 2020 for Abstracts checklist.	
**INTRODUCTION**			
Rationale	3	Describe the rationale for the review in the context of existing knowledge.	
Objectives	4	Provide an explicit statement of the objective(s) or question(s) the review addresses.	
**METHODS**			
Eligibility criteria	5	Specify the inclusion and exclusion criteria for the review and how studies were grouped for the syntheses.	
Information sources	6	Specify all databases, registers, websites, organisations, reference lists and other sources searched or consulted to identify studies. Specify the date when each source was last searched or consulted.	
Search strategy	7	Present the full search strategies for all databases, registers and websites, including any filters and limits used.	
Selection process	8	Specify the methods used to decide whether a study met the inclusion criteria of the review, including how many reviewers screened each record and each report retrieved, whether they worked independently, and if applicable, details of automation tools used in the process.	
Data collection process	9	Specify the methods used to collect data from reports, including how many reviewers collected data from each report, whether they worked independently, any processes for obtaining or confirming data from study investigators, and if applicable, details of automation tools used in the process.	
Data items	10a	List and define all outcomes for which data were sought. Specify whether all results that were compatible with each outcome domain in each study were sought (e.g. for all measures, time points, analyses), and if not, the methods used to decide which results to collect.	
	10b	List and define all other variables for which data were sought (e.g. participant and intervention characteristics, funding sources). Describe any assumptions made about any missing or unclear information.	
Study risk of bias assessment	11	Specify the methods used to assess risk of bias in the included studies, including details of the tool(s) used, how many reviewers assessed each study and whether they worked independently, and if applicable, details of automation tools used in the process.	
Effect measures	12	Specify for each outcome the effect measure(s) (e.g. risk ratio, mean difference) used in the synthesis or presentation of results.	

For peer review only - http://bmjopen.bmj.com/site/about/guidelines.xhtml

Section and Topic	Item #	Checklist item	Location where item is reported
Synthesis methods	13a	Describe the processes used to decide which studies were eligible for each synthesis (e.g. tabulating the study intervention characteristics and comparing against the planned groups for each synthesis (item #5)).	
	13b	Describe any methods required to prepare the data for presentation or synthesis, such as handling of missing summary statistics, or data conversions.	
	13c	Describe any methods used to tabulate or visually display results of individual studies and syntheses.	
	13d	Describe any methods used to synthesize results and provide a rationale for the choice(s). If meta-analysis was performed, describe the model(s), method(s) to identify the presence and extent of statistical heterogeneity, and software package(s) used.	
	13e	Describe any methods used to explore possible causes of heterogeneity among study results (e.g. subgroup analysis, meta-regression).	
	13f	Describe any sensitivity analyses conducted to assess robustness of the synthesized results.	
Reporting bias assessment	14	Describe any methods used to assess risk of bias due to missing results in a synthesis (arising from reporting biases).	
Certainty assessment	15	Describe any methods used to assess certainty (or confidence) in the body of evidence for an outcome.	
**RESULTS**			
Study selection	16a	Describe the results of the search and selection process, from the number of records identified in the search to the number of studies included in the review, ideally using a flow diagram.	
	16b	Cite studies that might appear to meet the inclusion criteria, but which were excluded, and explain why they were excluded.	
Study characteristics	17	Cite each included study and present its characteristics.	
Risk of bias in studies	18	Present assessments of risk of bias for each included study.	
Results of individual studies	19	For all outcomes, present, for each study: (a) summary statistics for each group (where appropriate) and (b) an effect estimate and its precision (e.g. confidence/credible interval), ideally using structured tables or plots.	
Results of syntheses	20a	For each synthesis, briefly summarise the characteristics and risk of bias among contributing studies.	
	20b	Present results of all statistical syntheses conducted. If meta-analysis was done, present for each the summary estimate and its precision (e.g. confidence/credible interval) and measures of statistical heterogeneity. If comparing groups, describe the direction of the effect.	
	20c	Present results of all investigations of possible causes of heterogeneity among study results.	
	20d	Present results of all sensitivity analyses conducted to assess the robustness of the synthesized results.	

For peer review only - http://bmjopen.bmj.com/site/about/guidelines.xhtml

Section and Topic	Item #	Checklist item	Location where item is reported
Reporting biases	21	Present assessments of risk of bias due to missing results (arising from reporting biases) for each synthesis assessed.	
Certainty of evidence	22	Present assessments of certainty (or confidence) in the body of evidence for each outcome assessed.	
**DISCUSSION**			
Discussion	23a	Provide a general interpretation of the results in the context of other evidence.	
	23b	Discuss any limitations of the evidence included in the review.	
	23c	Discuss any limitations of the review processes used.	
	23d	Discuss implications of the results for practice, policy, and future research.	
**OTHER INFORMATION**			
Registration and protocol	24a	Provide registration information for the review, including register name and registration number, or state that the review was not registered.	
	24b	Indicate where the review protocol can be accessed, or state that a protocol was not prepared.	
	24c	Describe and explain any amendments to information provided at registration or in the protocol.	
Support	25	Describe sources of financial or non-financial support for the review, and the role of the funders or sponsors in the review.	
Competing interests	26	Declare any competing interests of review authors.	
Availability of data, code and other materials	27	Report which of the following are publicly available and where they can be found: template data collection forms; data extracted from included studies; data used for all analyses; analytic code; any other materials used in the review.	

For peer review only

Appendix 3. PRISMA flowchart for new systematic reviews.

From: Page MJ, McKenzie JE, Bossuyt PM, Boutron I, Hoffmann TC, Mulrow CD, et al. The PRISMA 2020 statement: an updated guideline for reporting systematic reviews. BMJ 2021;372:n71. doi: 10.1136/bmj.n71

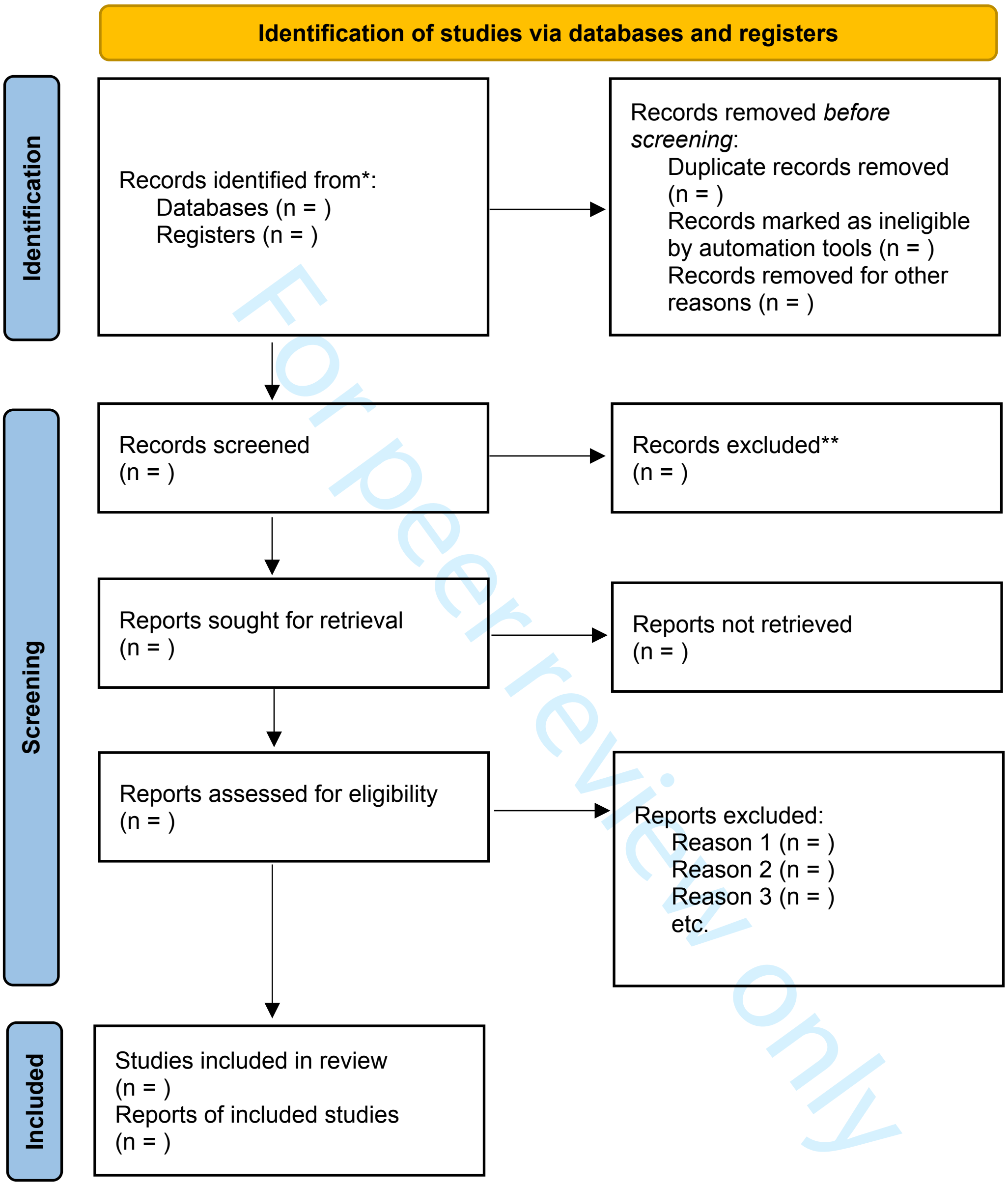

For peer review only

*Consider, if feasible to do so, reporting the number of records identified from each database or register searched (rather than the total number across all databases/registers).

**If automation tools were used, indicate how many records were excluded by a human and how many were excluded by automation tools.

For peer review only - http://bmjopen.bmj.com/site/about/guidelines.xhtml

Appendix 4. PRISMA flowchart for updated systematic reviews.

From: Page MJ, McKenzie JE, Bossuyt PM, Boutron I, Hoffmann TC, Mulrow CD, et al. The PRISMA 2020 statement: an updated guideline for reporting systematic reviews. BMJ 2021;372:n71. doi: 10.1136/bmj.n71

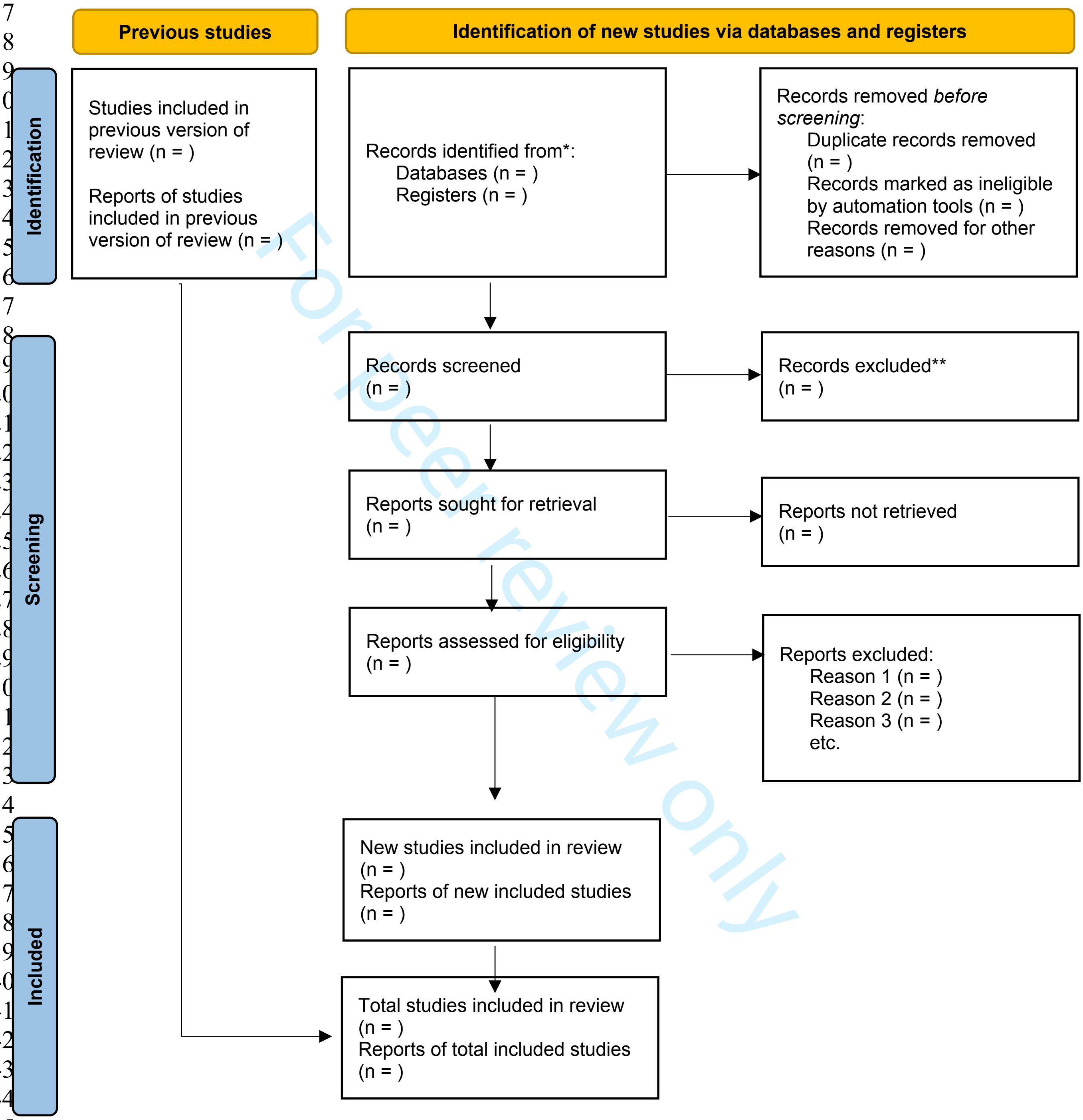

*Consider, if feasible to do so, reporting the number of records identified from each database or register searched (rather than the total number across all databases/registers).

**If automation tools were used, indicate how many records were excluded by a human and how many were excluded by automation tools.

For peer review only

BMJ Open

The reporting quality of published reviews of commercial and publicly available mobile health apps (mHealth app reviews): A scoping review protocol

Journal:	*BMJ Open*
Manuscript ID	bmjopen-2023-083364.R1
Article Type:	Protocol
Date Submitted by the Author:	22-Mar-2024
Complete List of Authors:	Gasteiger, Norina; The University of Manchester, Division of Nursing, Midwifery and Social Work; NIHR Applied Research Collaboration Greater Manchester<br>Norman, Gill; The University of Manchester, Division of Nursing, Midwifery & Social Work; NIHR Applied Research Collaboration Greater Manchester<br>Grainger, Rebecca; University of Otago, Department of Medicine<br>Eost-Telling, Charlotte; The University of Manchester, School of Nursing, Midwifery & Social Work; NIHR Applied Research Collaboration Greater Manchester<br>Jones, Debra; The University of Manchester, School of Nursing, Midwifery & Social Work<br>Ali, Syed; The University of Manchester, Centre for Health Informatics, Division of Informatics, Imaging & Data Sciences, Manchester Academic Health Science Centre; NIHR Applied Research Collaboration Greater Manchester<br>van der Veer, Sabine N.; The University of Manchester, Centre for Health Informatics, Division of Informatics, Imaging & Data Sciences, Manchester Academic Health Science Centre; NIHR Applied Research Collaboration Greater Manchester<br>Ford, Claire; The University of Manchester, Division of Nursing, Midwifery & Social Work<br>Hall, Alex; The University of Manchester, Division of Nursing, Midwifery & Social Work<br>Law, Kate; The University of Manchester, Division of Nursing, Midwifery & Social Work; The Christie Hospital NHS Trust<br>Byerly, Matthew; The University of Kansas School of Medicine<br>Davies, Alan; The University of Manchester, Centre for Health Informatics, Division of Informatics, Imaging & Data Sciences, Manchester Academic Health Science Centre<br>Paripoorani, Deborah; Manchester Royal Infirmary, EMERGING Research Team; NIHR Applied Research Collaboration Greater Manchester<br>Shi, Chunhu; The University of Manchester, Division of Nursing, Midwifery & Social Work; NIHR Applied Research Collaboration Greater Manchester<br>Dowding, Dawn; The University of Manchester, Division of Nursing, Midwifery & Social Work; NIHR Applied Research Collaboration Greater Manchester

**Primary Subject Heading**:	Research methods
Secondary Subject Heading:	Health informatics
Keywords:	Information technology < BIOTECHNOLOGY & BIOINFORMATICS, Telemedicine < BIOTECHNOLOGY & BIOINFORMATICS, eHealth, STATISTICS & RESEARCH METHODS

SCHOLARONE™ Manuscripts

The reporting quality of published reviews of commercial and publicly available mobile health apps (mHealth app reviews): A scoping review protocol

Norina Gasteiger[1,2], Gill Norman[1,2,3], Rebecca Grainger[4], Charlotte Eost-Telling[1,2], Debra Jones[1], Syed Mustafa Ali[2,5], Sabine N. van der Veer[2,5], Claire R. Ford[1], Alex Hall[1], Kate Law[1,6], Matthew Byerly[7], Alan Davies[5], Deborah Paripoorani[2,8], Chunhu Shi[1,2] & Dawn Dowding[1,2]

1. Division of Nursing, Midwifery and Social Work, University of Manchester, Manchester, UK
2. National Institute for Health and Care Research Applied Research Collaboration Greater Manchester, Manchester, UK
3. NIHR Innovation Observatory, Population Health Sciences Institute, Newcastle University, Newcastle, UK
4. Department of Medicine, University of Otago Wellington, Wellington, New Zealand
5. Centre for Health Informatics, Division of Informatics, Imaging and Data Sciences, Manchester Academic Health Science Centre, University of Manchester, Manchester, UK
6. The Christie Hospital NHS Foundation Trust, Manchester, UK
7. The University of Kansas School of Medicine-Wichita, Wichita, Kansas, United States of America
8. EMERGING Research Team, Manchester Royal Infirmary, Manchester, UK

For peer review only - http://bmjopen.bmj.com/site/about/guidelines.xhtml

AUTHOR DETAILS

	ORCiD	Email address
Dr Norina Gasteiger	0000-0001-7801-7417	norina.gasteiger@manchester.ac.uk
Dr Gill Norman	0000-0002-3972-5733	gill.norman@manchester.ac.uk
Professor Rebecca Grainger	0000-0001-9201-8678	rebecca.grainger@otago.ac.nz
Dr Charlotte Eost-Telling	0000-0002-9568-3195	charlotte.eost-telling@manchester.ac.uk
Mrs Debra Jones	0000-0003-1479-3622	debra.jones@manchester.ac.uk
Mr Syed Mustafa Ali	0000-0001-9393-9049	syedmustafa.ali@manchester.ac.uk
Dr Sabine N. van der Veer	0000-0003-0929-436X	sabine.vanderveer@manchester.ac.uk
Dr Claire R. Ford	0000-0003-2950-1565	claire.ford@manchester.ac.uk
Dr Alex Hall	0000-0002-8849-744X	alex.hall@manchester.ac.uk
Ms Kate Law	0000-0002-0995-8309	kate.law@postgrad.manchester.ac.uk
Professor Matthew Byerly	0000-0001-7033-1268	mbyerly3@kumc.edu
Dr Alan Davies	0000-0001-5737-5629	alan.davies-2@manchester.ac.uk
Ms Deborah Paripoorani	N/A	Deborah.Paripoorani@mft.nhs.uk
Dr Chunhu Shi	0000-0003-0151-0451	chunhu.shi@manchester.ac.uk
Professor Dawn Dowding	0000-0001-5672-8605	dawn.dowding@manchester.ac.uk

For peer review only

ABSTRACT

Introduction

Reviews of commercial and publicly available smartphone (mobile) health applications (mHealth app reviews) are being undertaken and published in the peer-reviewed literature. However, there is variation in the conduct and reporting of mHealth app reviews, with no existing reporting guidelines. We aim to develop a PRISMA extension to guide the conduct and reporting of mHealth app reviews. This scoping review of published mHealth app reviews will explore their alignment, deviation, and modification to the PRISMA 2020 items for systematic reviews and identify a list of possible items to include in a PRISMA-App extension reporting guideline.

Method and analysis

We are following the Joanna Briggs Institute approach and Arksey and O'Malley's five-step process. Patient and public contributors, mHealth app review, digital health research and evidence synthesis experts, healthcare professionals and a specialist librarian gave feedback on the methods. We will search SCOPUS, CINAHL Plus, AMED, EMBASE, Medline, APA PsycINFO and ACM Digital Library for articles reporting mHealth app reviews and use a two-step screening process to identify eligible articles. Information on whether the authors have reported, or how they have modified the PRISMA 2020 items in their reporting will be extracted. Data extraction will also include the article characteristics, protocol and registration information, review question frameworks used, information about the search and screening process, how apps have been evaluated, and evidence of stakeholder engagement. This will be analysed using a content synthesis approach and presented using descriptive statistics and summaries.

Ethics and dissemination

Ethical approval is not required. The findings will be disseminated through peer-reviewed journal publications (shared on our project website and on the EQUATOR Network website where the PRISMA-App extension has been registered as under development), conference presentations and blog and social media posts in lay language.

Registration

This protocol is registered on OSF (https://osf.io/5ahjx).

For peer review only

KEYWORDS

app review; mHealth; smartphone; research methods; reporting; scoping review

STRENGTHS AND LIMITATIONS

- This review will be conducted systematically, with data extraction informed by the PRISMA 2020 reporting items and a previous scoping review.
- The protocol has had input from a multidisciplinary team of mHealth app review, digital health and evidence synthesis experts, healthcare professionals, a librarian, and patient and public contributors.
- The broad scope of health topics to be included in the review increases the generalisability of the findings.
- In line with scoping review guidance, a quality appraisal of the included studies will not be conducted.
- Only mHealth app reviews reported in English will be included, meaning some other relevant reviews published in other languages may not be included.

WORD COUNT: 2,789

For peer review only

INTRODUCTION

In 2021 it was recorded that there are more than 350,000 health applications (apps) (1). These applications are increasingly being integrated into healthcare; supporting professionals in their clinical practice (2) and empowering patients to manage and monitor their health conditions (3, 4). However, the quality and reliability of mobile health (mHealth) apps varies significantly (5), as developers can release smartphone health apps without any evaluation, meaning it is a challenge for health professionals and users without expertise in health research and digital technology to identify and evaluate the suitability of mHealth apps for their use.

This has led to the emergence of a new method: systematic reviews of commercial and publicly available mHealth apps (hereafter called mHealth app reviews). This provides a standard approach to identify mHealth apps relevant to a particular use case and assess aspects such as quality and functionality. Many mHealth app reviews have now been published, for varied topics including genetics (6, 7), patient-reported outcomes in oncology (8), mental health (9, 10), rheumatoid arthritis (11), strength training (12), menopause (13), exercise (14, 15), hand hygiene (16), atrial fibrillation (17), pain (18-20) and smoking cessation (21, 22). These reviews can serve as a valuable resource for healthcare decision-makers, practitioners, patients and the general public seeking high-quality mHealth apps; can identify gaps in the field and may guide researchers and industry in developing new products.

While mHealth app reviews share features with traditional systematic literature reviews, they differ substantially in their methods and reporting (23) due to the review being of commercial and publicly available products on app stores, instead of published literature. Examples of traditional systematic reviews of literature describing apps include those on monitoring and managing mental health symptoms (24) and self-managing pregnancy (25). While literature reviews can tell us about the effectiveness of apps which have been evaluated and the results published, they do not provide a comprehensive overview of all apps that are commercially or publicly available for use by patients, healthcare professionals and the public. mHealth app reviews also differ as there are no formal requirements for the protocol to be registered, searches take place on app libraries, screening often takes place on Excel (rather than using specifically designed tools like Rayyan (26) or Covidence (27)), and they are more challenging to replicate, as apps may emerge, disappear or be updated between searches (23).

The EQUATOR Network (28) provides an array of guidelines for reporting evaluations of digital technologies, such as the CONSORT-EHEALTH checklist (an extension of the CONSORT checklist tailored for reporting randomised controlled trials of web-based and mobile health interventions (29)), and guidance on reporting evaluations of specific technologies, such as sensors (30), mHealth interventions (31), telehealth in clinical trials (32) and smartphone-delivered ecological momentary assessments (33). There are also several extensions of the Preferred Reporting Items for Systematic Reviews and Meta-Analyses (PRISMA) reporting guidelines (34) available for different types of literature reviews.

In contrast, no reporting guidelines exist for mHealth app reviews, and we are not aware of any currently in development. The need for a reporting standard for health app-focused reviews was emphasised in a scoping review published in 2020 (35). The authors reviewed 26 mHealth app reviews published between 2012 and 2018 and found issues in their reporting. For example, the date of the searches was sometimes unclear (38%, 10/26) or absent (15%, 4/26). The number of reviewers

For peer review only - http://bmjopen.bmj.com/site/about/guidelines.xhtml

involved was also poorly reported in 58% of reviews, and in 83% it was unclear whether screening was performed independently. Another important finding was the absence of clinical recommendations or reporting on clinical efficacy, found in 77% of the included reviews. Adhering to reporting guidelines may help to minimise the presence of these inconsistencies in reporting, and ensure that standardised information is presented.

If a key purpose of an mHealth app review is for people to be able to identify the best product for a particular purpose, it is important to further explore how app review authors have evaluated the apps and reported on their outcomes. For example, some previous app reviews have considered the accessibility of the apps by generating readability metrics on the written content (13, 36, 37). This is especially important for patient or public-facing apps. There are also various approaches to reporting on efficacy (i.e., that the app results in intended outcomes). This may include searching for evidence of a previous evaluation within the app itself, on developers' websites, or for published literature or conference abstracts in academic databases. For example the Mobile App Rating Scale (MARS) (38) is commonly used in mHealth app reviews and has an item which addresses *whether an app has been trialled or tested.* Previous examples of app reviews have approached this item by excluding the item entirely (11) or searching Google Scholar (6, 8, 13, 16, 18). It is unclear how other authors have evaluated the apps and how this has informed their recommendations of apps as being high-quality. Understanding the nature of the evaluations (especially efficacy evidence) seems essential in mHealth app reviews as readers, including health and care workers, patients and the public, and healthcare decision-makers may use these to choose which apps to use.

We have previously discussed the methodological considerations for conducting systematic mHealth app reviews; introducing a 7-step method and the TECH framework (Target user, Evaluation focus, Connectedness and the Health domain) for developing research (review) questions and determining app eligibility criteria (23). This is the first stage of a broader project that aims to systematise the process of conducting and reporting mHealth app reviews.

The next step is to develop reporting guidelines to support authors of mHealth app reviews in transparently presenting their methods and findings. The field of app reviews is rapidly developing and expanding, so a new scoping review is required to update that previously reported, which had a search date of 2018 (35). A preliminary search of the Cochrane Database of Systematic Reviews, Google Scholar and JBI Evidence Synthesis was conducted, and no current or ongoing systematic reviews or scoping reviews on the topic were identified. Therefore, we are undertaking a scoping review to build on and update the scoping review by Grainger et al. (35).

OBJECTIVES

In line with guidance for developing reporting guidelines (39), the next step is to search for relevant evidence on the quality of the reporting of published mHealth app reviews. The aim of this work is, therefore, to conduct a scoping review on published mHealth app reviews to explore their alignment, deviation and modification to the PRISMA 2020 items and identify a list of possible items to include in a new PRISMA-App review reporting guideline extension.

METHODS

Scoping review

For peer review only - http://bmjopen.bmj.com/site/about/guidelines.xhtml

The methods for this scoping review were developed in alignment with the Joanna Briggs Institute approach for scoping reviews (40) and reviewed by a group of patient and public contributors and an advisory group consisting of mHealth app review, digital health research and evidence synthesis experts and NHS healthcare professionals interested in app reviews. The review will be carried out using the five-step process for conducting scoping reviews, originally outlined by Arksey and O'Malley (41). This protocol has already been registered and made publicly available on OSF (https://osf.io/5ahjx).The review will be reported using the PRISMA-ScR extension guidelines for scoping reviews (42) (see Appendix 1). We will start the review on the 2nd January 2024 and complete it by the 2nd September, 2024.

Procedures

Identifying the initial research question

We used the Study, Data, Methods and Outcomes (SDMO) acronym to inform our research questions and eligibility criteria, which has been recommended when conducting reviews on methodology or theory (43). Additionally, as suggested by Levac et al. (44), we considered the purpose and expected outputs of the review to assist in writing the research questions. The purpose and expected outputs are primarily a list of potential reporting items used to inform the future PRISMA-App review guideline. Focussing on the PRISMA 2020 (34) items is appropriate as many app review authors already informally use the PRISMA items to report their work or have attempted to amend the PRISMA flowchart when reporting their app search and screening process (6, 8, 11-13, 16-18, 21).

The second question seeks to understand what outcomes were evaluated in mHealth app reviews (e.g., usability, functionality, privacy, accessibility and efficacy). This builds on the previous review (35), which found that most of the app reviews did not make clinical recommendations or report clinical efficacy (i.e., whether the app could meet desired outcomes in a clinical context). We are therefore interested in understanding what the outcomes were in general, and whether any of the app reviews reported on efficacy in any other sense such as satisfaction, increased knowledge or perceived support.

The two key questions are:

1. In published reviews of commercial and publicly available mHealth apps how does reporting align with or deviate from the PRISMA 2020 items? Have authors used items that directly align with PRISMA 2020 items or have these been modified?
2. What outcomes did the mHealth app reviews evaluate and report on?

Identifying relevant studies

We will search the SCOPUS, CINAHL Plus (via EBSCO), AMED (via Ovid), EMBASE (via Ovid), Medline (via Ovid), APA PsycINFO and ACM Digital Library databases for published mHealth app reviews, under the guidance of a teaching and learning librarian who has given input on the search strategy. Reference lists of eligible articles will also be hand-searched for additional sources (snowballing), while a forward citation approach will be used to identify app reviews that have cited earlier published work.

For peer review only - http://bmjopen.bmj.com/site/about/guidelines.xhtml

The key terms used to build the search strategy are shown in Table 1, with the full search strategy presented in Appendix 2. Where appropriate, subject headings will be applied in the databases. The keywords will be separated by the 'OR' Boolean operator. The technology and review type keywords will be separated by the 'AND' Boolean operator. The proximity function using five words will be used for the review type keywords, to include different variations of app review, such as review of apps, review of smartphone apps, or review of patient-facing mobile health applications.

Publication date will be limited from 1st January 2007, as the first iPhone (and first smartphone) was introduced on 29th June 2007, so there will be no apps available to review before 2007.

Table 1. Keywords used to identify literature.

Technology	Review type
• Mobile<br>• Smartphone<br>• "Cell phone"<br>• mHealth<br>• "mobile health"<br>• eHealth<br>• Tele* (to include telehealth, telemedicine and other variations)	(App or apps or application or applications) adj5[1] review

Note:
[1]The exact wording depends on the database and may include adj5, W/5 or N5. The ACM Digital Library does not allow the proximity function, so "app* review" will be used instead.

Study selection

Table 2 presents the eligibility criteria for the literature, using the SDMO acronym (43). Broadly, our inclusion criteria are:

- Reviews of commercial and publicly available mobile (smartphone) apps published in English and after 1 January 2007 (types of study)
- that have a health focus (type of data),
- include any method and measure of evaluating apps (e.g., MARS or user ratings) and,
- have any outcome or focus, including focussing on the availability of apps or evaluation (e.g., quality, functionality, privacy and security or adherence to clinical guidelines).

We will only include mHealth app reviews published in English. However, we will separately list articles that were excluded due to language, which can enable others to easily identify these papers for subsequent reviews.

For peer review only - http://bmjopen.bmj.com/site/about/guidelines.xhtml
For peer review only

Table 2. Eligibility criteria for the mHealth app reviews to be included in the scoping review.

**SDMO Acronym**	**Inclusion criteria**	**Exclusion criteria**	**No limits**
Types of study	Reviews of commercial and publicly available mobile apps<br>• *Must be focussed on smartphone (mobile) apps.*<br>• *Can be identified as systematic, scoping or without a specific approach named.*<br>• *Some app reviews may be combined with other literature reviews or reviews of other apps. These will only be included if detail is reported separately on the smartphone app review methods and results.*<br>• *Reviews including other technology (e.g., iPads, digital assistants, virtual reality headsets or smartwatches) will only be included if the focus is on smartphone apps and the other technology is used only to operationalise some of the functions.*<br><br>English language<br><br>Published on or after 1 January, 2007	Literature reviews<br><br>Reviews of other technology or apps (e.g., websites, computer apps, iPad apps)<br><br>Full-text not available<br>• *Exclude abstracts and documents where there is insufficient information or the full-text is not available.*<br><br>Not in English<br><br>Published before 1 January, 2007	Document type<br>• *Any document type will be included, if there is a full-text available so that enough information can be extracted (e.g., full-length conference papers, journal articles, book chapters).*<br><br>Smartphone device<br><br>Operating system requirements<br><br>App markets<br><br>Geography (location)
Types of data	Health focus<br><br>*Must be focussed on a health topic, whereby the apps are marketed for physical or mental health or general well-being. This may include (but is not limited to) apps that educate, empower or inform users on a health topic (e.g., genetics), self-monitor/manage or change health behaviours (e.g., sleep, nutrition, exercise or smoking cessation) or are used for social support or in health systems or by patients, administrators and health and care workers or decision-makers (e.g., screening, diagnosis, triage, appointment-booking, remote monitoring, decision-making, training and treatment).*	Not focussed on health topics	Health topic<br>• *Apps can be for any health topic.*<br><br>Intended users of apps<br>• *Apps can be for any stakeholder, including patients, the public, health professionals and the health system.*
Types of methods	Any method (and measure) of evaluating apps can be included, such as using validated measures (e.g., MARS (38)), synthesising content presented within the app or user ratings and reviews on app markets.	N/A	App evaluation measures and methods.
Types of outcomes	Any outcome, including those related to evaluating app quality, functionality, privacy and security, accessibility or efficacy. This would also include app reviews that simply focus on identifying which apps were available, summarised their content or described the extent to which they adhere to best clinical practice/guidelines.	N/A	Any outcomes.

For peer review only

For peer review only - http://bmjopen.bmj.com/site/about/guidelines.xhtml

The final search results will be imported into Rayyan (26) for deduplication and screening. As suggested by Levac et al. (44), the screening process will be iterative and use a team-based approach. An initial meeting will be held with all researchers involved in the screening process to discuss interpretation of the eligibility criteria and reach shared understanding after an initial set of records have been pilot screened. A two-step process will then be followed. First, two researchers will independently review each abstract/title against the eligibility criteria. Second, the full text of records potentially eligible based on abstract/title screening will be reviewed by two researchers independently. A meeting will be held after the second stage to discuss and reach consensus where there is disagreement. A third reviewer will make the final decision if consensus cannot be reached. Depending on the number of articles, two teams of two researchers may perform the screening process, with a fifth researcher available to resolve disagreements. The search and screening process will be presented as a flowchart.

Charting the data

A data extraction sheet will be created in Excel using headers related to our 7-step method and TECH framework (23), the PRISMA 2020 items (34), whether and how authors modified them, additional information reported, and methods used to appraise the apps' quality, functionality or efficacy. Some items have also been taken from the review conducted by Grainger et al. (35) as these capture details unique to app reviews (e.g., how app stores were searched). Table 3 presents the data extraction items.

Table 3. Information to be extracted from the articles.

**Article characteristics**	
Title	• Title of the article.<br>• Does it name a review method in the title (e.g., systematic, scoping, app review)?
Date	• Year of publication.
Journal	• Name of the journal the app review is published in.<br>• Name and contact information of the editor-in-chief.[1]
Authors	• Name and contact information of all authors.[1]<br>• Source of funding (if any).
Objective	• Aim or research question.
Topic and context	• The health problem being explored.<br>• App target user.<br>• Context: where the app is to be used, including location of care (acute, primary health care, community, long-term care, etc.) and geographical location.
**Protocol and registration**	
A priori review registration	• Was the study protocol registered and was the protocol available?[2]
**Review question**	
Review question frameworks	• Was a framework used to write the review question (e.g., PICO)? If so, which?<br>• Did this align with any of the TECH components? If so, which?
**Reporting guidelines**	
Reporting guidelines	• Did authors state which reporting guideline they used? If so, which?<br>• Did the authors clearly mention amending any guidelines?
Alignment, deviation and modification to the PRISMA (2020) items (see Appendix 3)	• For each of the PRISMA (2020) items, identify whether the information was reported as is, or how this was modified (if applicable).
**Search and screening**	

For peer review only

For peer review only - http://bmjopen.bmj.com/site/about/guidelines.xhtml

Flow charts/diagrams	• Did the authors present a flow diagram for the search and screening process?<br>• Did the authors report a PRISMA (2020) flow chart for new or updated systematic reviews (see Appendix 4 and 5)?<br>• Were amendments made to the PRISMA (2020) flow charts (see Appendix 4 and 5)? If yes, which?
App store search	• Were the apps store(s) searched described including the a) keywords, b) countries/location and c) a clear description of dates of the search?[2]<br>• Was the method for identifying and removing duplicate apps clearly described, including metadata (e.g., version numbers) used to determine if apps were duplicates?[2]<br>• Were the number and independence of people screening apps described?[2]<br>• Were limits on inclusion of apps based on other factors clearly described (lite or full version, paid or free versions, non-English)?[2]<br>• Where the same app featured in different app stores (multiplatform apps), was there a clear statement of which apps were included?[2]<br>• When apps were downloaded to phones for data extraction, were the phone model and version of the operating system clearly reported?[2]
**Evaluating the apps and making recommendations**	
Outcomes	• Which outcomes did the review evaluate? e.g., quality, functionality, usability, privacy, efficacy, accessibility or other
Quality, functionality, usability and other assessments	• Was a best practice content tool (e.g., clinical guideline) used to evaluate app content or quality? Was the source identified, were any modifications made described, and was the use of the guideline justified?[2]<br>• Was an app quality, functionality and/or usability evaluation undertaken using established measures and frameworks (e.g., MARS)?<br>• Are any other tools/instruments used to evaluate app quality and/or usability, and was the source of these described?[2]<br>• Were security and privacy considered? If so, what aspects were included? e.g., login, password, privacy policy, access to microphone or camera, encryption or data sharing.
Efficacy	• Did the authors report on the efficacy of the apps?<br>• If yes, where did they obtain this information and what methods were used?
Accessibility	• Was the accessibility of the apps evaluated, considering different needs of target users?
Recommendations	• Were any apps recommended for use in the clinical setting or by people with chronic health conditions?[2]<br>• Were any apps recommended overall? What informed this recommendation?
**Stakeholder engagement or consultation**	
Patient, public and expert engagement	• Were any patients or members of the public involved in the app review?<br>• Were any other stakeholders involved or consulted (e.g., industry partners, clinicians or software developers)? If so, which?<br>• Was a lay summary provided? If so, were any apps clearly recommended?
**Other**	
Other domains	• Any other domains reported on? If yes, what and how?

Notes:

[1] This information will not be published publicly as part of the review. It will be used to create a database of authors of mHealth app reviews and journals who have published them. This is required for the next step of the

For peer review only - http://bmjopen.bmj.com/site/about/guidelines.xhtml

project- the Delphi (consensus-building) study, as mHealth experts will be participants and potential partners in this process.

[2] These data extraction items were taken from the scoping review conducted by Grainger et al. (35).

Consistent with recommendations by Levac et al. (44), we will take an iterative approach to charting by continually updating the data-charting form as needed. The research team will first pilot the data extraction sheet, by extracting the data from one app review, with discussion afterward to ensure consistency in interpretation of the items. Data will then be extracted from the other articles, with one author extracting the information and another checking this. Depending on the final number of included articles, this will be split between the researchers.

It has been suggested that some scoping reviews should also include quality assessments of the methodology used in the articles (44). However, as this is not the focus of our review and as no specific quality assessment tool currently exists for mHealth app reviews, the quality of the included studies will not be assessed.

Collating, summarising, and reporting the results

Similar to the previous review (35) and as recommended by Arksey and O'Malley (41), we will report data as frequencies (where possible) to determine which items were reported as is, or whether they were modified.

Information that cannot be reported as frequencies, on how the PRISMA 2020 items were modified and how other relevant information was reported will be summarised using a content synthesis approach, to help inform amendments to the existing guideline and to identify new items.

The results overall will be reported using descriptions and examples, while some of the numerical results will also be presented using tables and figures.

Strengths and limitations

This scoping review will be conducted in a systematic and rigorous manner, with data extraction informed by the existing PRISMA 2020 (34) reporting items and a previous scoping review (35). It also adheres to existing guidance on conducting scoping reviews, including from the Joanna Briggs Institute (40) and Arksey and O'Malley (41), and has had input from a multidisciplinary team of mHealth app review, digital health and evidence synthesis experts, NHS healthcare professionals, a librarian and patient and public contributors. The breadth of its scope of health topics (and methodological focus), also means that the findings will be widely generalisable.

A fundamental limitation is the inability to assess the quality of the included reviews, due to an absence of quality appraisal tools for reviews of commercial and publicly available mHealth apps. This limitation means that low quality app reviews may also contribute to the development of the future PRISMA-App reporting guideline. However, we will mitigate this in the next phase of the project, in which a Delphi study with experts will help to prioritise the items. Another limitation concerns restricting the included mHealth app reviews to those reported in English which may lead to other relevant reviews being excluded.

For peer review only - http://bmjopen.bmj.com/site/about/guidelines.xhtml

Patient and public involvement

We have established a PPIE group to give feedback on all aspects of our project. The group has been consulted to provide input on the protocol and suggested additional items that should be extracted (i.e., whether the accessibility of the apps was evaluated and whether a lay summary was provided). They also gave ideas for how the findings should be disseminated. We will continue to consult with them throughout the review; this will inform the iterative aspects of the scoping review process and will help to guide the findings and their dissemination.

ETHICS AND DISSEMINATION

Ethical approval is not required to conduct this scoping review which will use only previously published data.

The findings of this scoping review will be disseminated through peer-reviewed journal publications which will be shared on our project website and on the EQUATOR Network website where the PRISMA-App review extension has been registered as under development, in addition to conference presentations and blog posts and short summaries in lay language on professional social media.

CONCLUSION

This protocol describes how we will conduct a scoping review on published mHealth app reviews to explore their alignment, deviation and modification to the PRISMA 2020 items and identify a list of possible items to include in the new PRISMA-App review reporting guideline extension. The results will inform the next phase in developing the new PRISMA-App review reporting guideline: a Delphi study to reach a consensus on which items are most relevant and important to include in the guideline.

DECLARATIONS

Author contributions

NG, GN and DD designed the protocol. NG wrote the first draft of the manuscript, with support from GN. NG, GN, RG, CE-T, DJ, SMA, SNvdV, CRF, AH, KL, MB, AD, DP, CS and DD revised the protocol critically and approved the final manuscript.

Funding

This work is funded by the National Institute for Health and Care Research Applied Research Collaboration Greater Manchester (Grant award: NIHR200174). The views expressed in this publication are those of the authors and not necessarily those of the National Institute for Health and Care Research or the Department of Health and Social Care.

Acknowledgements

We would like to acknowledge Mr Michael Stevenson for assisting with the search strategy. We would also like to thank our patient and public contributors for their input- thank you Amber McAvoy, Eric Lowndes, Ashgan Mahyoub and Beatrice Namu. We acknowledge Mrs Amy Vercell and Dr Lisa McGarrigle who are part of our team.

For peer review only

Competing interests

The authors have no competing interests to declare.

Patient consent for publication

Not required.

Data availability

No new data has been generated for this protocol.

References

[1] IQVIA. Digital Health Trends 2021: Innovation, evidence, regulation and adoption. [Internet]. 2021. Available from: https://www.iqvia.com/insights/the-iqvia-institute/reports-and-publications/reports/digital-health-trends-2021

[2] Mayer MA, Rodríguez Blanco O and Torrejon A. Use of Health Apps by Nurses for Professional Purposes: Web-Based Survey Study. *JMIR Mhealth Uhealth.* 2019;7(11):e15195. 2019/11/05. doi: 10.2196/15195.

[3] Dsouza SM, Shetty S, Venne J, et al. Effectiveness of self-management applications in improving clinical health outcomes and adherence among diabetic individuals in low and middle-income countries: a systematic review. *BMJ Open.* 2022;12(11):e060108. 2022/11/16. doi: 10.1136/bmjopen-2021-060108.

[4] Doumen M, De Cock D, Van Lierde C, et al. Engagement and attrition with eHealth tools for remote monitoring in chronic arthritis: a systematic review and meta-analysis. *RMD Open.* 2022;8(2) 2022/10/28. doi: 10.1136/rmdopen-2022-002625.

[5] Larsen ME, Huckvale K, Nicholas J, et al. Using science to sell apps: Evaluation of mental health app store quality claims. *NPJ Digit Med.* 2019;2:18. 2019/07/16. doi: 10.1038/s41746-019-0093-1.

[6] Gasteiger N, Vercell A, Davies A, et al. Patient-facing genetic and genomic mobile apps in the UK: a systematic review of content, functionality, and quality. *J Community Genet.* 2022;13(2):171-182. 2022/02/20. doi: 10.1007/s12687-022-00579-y.

[7] Talwar D, Yeh Y, Chen W-J, et al. Characteristics and quality of genetics and genomics mobile apps: a systematic review. *Eur J Hum Genet.* 2019;27:833–840. doi: https://doi.org/10.1038/s41431-019-0360-2.

[8] Vercell A, Gasteiger N, Yorke J, et al. Patient-facing cancer mobile apps that enable patient reported outcome data to be collected: A systematic review of content, functionality, quality, and ability to integrate with electronic health records. *Int J Med Inform.* 2022;170:104931. 2022/12/04. doi: 10.1016/j.ijmedinf.2022.104931.

[9] Martín-Martín J, Muro-Culebras A, Roldán-Jiménez C, et al. Evaluation of Android and Apple Store Depression Applications Based on Mobile Application Rating Scale. *Int J Environ Res Public Health.* 2021;18(23) 2021/12/11. doi: 10.3390/ijerph182312505.

[10] Tsai Z, Kiss A, Nadeem S, et al. Evaluating the effectiveness and quality of mobile applications for perinatal depression and anxiety: A systematic review and meta-analysis. *Journal of Affective Disorders.* 2022;296:443-453. doi: https://doi.org/10.1016/j.jad.2021.09.106.

[11] Grainger R, Townsley H, White B, et al. Apps for People With Rheumatoid Arthritis to Monitor Their Disease Activity: A Review of Apps for Best Practice and Quality. *JMIR Mhealth Uhealth.* 2017;5(2):e7. doi: 10.2196/mhealth.6956.

[12] McGarrigle L, Boulton E and Todd C. Map the apps: a rapid review of digital approaches to support the engagement of older adults in strength and balance exercises. *BMC Geriatr.* 2020;20(1):483. 2020/11/20. doi: 10.1186/s12877-020-01880-6.

For peer review only

[13] Paripoorani D, Gasteiger N, Hawley-Hague H, et al. A systematic review of menopause apps with an emphasis on osteoporosis. *BMC Womens Health.* 2023;23(1):518. 2023/09/29. doi: 10.1186/s12905-023-02612-9.

[14] Knight E, Stuckey MI, Prapavessis H, et al. Public Health Guidelines for Physical Activity: Is There an App for That? A Review of Android and Apple App Stores. *JMIR mHealth uHealth.* 2015;3(2):e43. doi: 10.2196/mhealth.4003.

[15] Payne HE, Moxley VB and MacDonald E. Health Behavior Theory in Physical Activity Game Apps: A Content Analysis. *JMIR Serious Games.* 2015;3(2):e4. 2015/07/15. doi: 10.2196/games.4187.

[16] Gasteiger N, Dowding D, Ali SM, et al. Sticky apps, not sticky hands: A systematic review and content synthesis of hand hygiene mobile apps. *J Am Med Inform Assoc.* 2021;28(9):2027-2038. 2021/06/29. doi: 10.1093/jamia/ocab094.

[17] Pearsons A, Hanson CL, Gallagher R, et al. Atrial fibrillation self-management: a mobile telephone app scoping review and content analysis. *Eur J Cardiovasc Nurs.* 2021;20(4):305-314. 2021/02/24. doi: 10.1093/eurjcn/zvaa014.

[18] Ali SM, Lau WJ, McBeth J, et al. Digital manikins to self-report pain on a smartphone: A systematic review of mobile apps. *Eur J Pain.* 2021;25(2):327-338. 2020/10/29. doi: 10.1002/ejp.1688.

[19] Kheirinejad S, Visuri A, Suryanarayana SA, et al. Exploring mHealth applications for self-management of chronic low back pain: A survey of features and benefits. *Heliyon.* 2023;9(6):e16586. 2023/06/22. doi: 10.1016/j.heliyon.2023.e16586.

[20] Devan H, Farmery D, Peebles L, et al. Evaluation of Self-Management Support Functions in Apps for People With Persistent Pain: Systematic Review. *JMIR Mhealth Uhealth.* 2019;7(2):e13080. 2019/02/13. doi: 10.2196/13080.

[21] Seo S, Cho SI, Yoon W, et al. Classification of Smoking Cessation Apps: Quality Review and Content Analysis. *JMIR Mhealth Uhealth.* 2022;10(2):e17268. 2022/02/18. doi: 10.2196/17268.

[22] Chen J, Chu J, Marsh S, et al. Smartphone App-Based Interventions to Support Smoking Cessation in Smokers with Mental Health Conditions: A Systematic Review. *Psych* 5: 1077-1100. DOI: 10.3390/psych5040072.

[23] Gasteiger N, Dowding D, Norman G, et al. Conducting a systematic review and evaluation of commercially available mobile applications (apps) on a health-related topic: the TECH approach and a step-by-step methodological guide. *BMJ Open.* 2023;13(e073283). doi: 10.1136/bmjopen-2023-073283.

[24] Wang K, Varma DS and Prosperi M. A systematic review of the effectiveness of mobile apps for monitoring and management of mental health symptoms or disorders. *Journal of Psychiatric Research.* 2018;107:73-78. doi: https://doi.org/10.1016/j.jpsychires.2018.10.006.

[25] Iyawa GE, Dansharif AR and Khan A. Mobile apps for self-management in pregnancy: a systematic review. *Health and Technology.* 2021;11(2):283-294. doi: 10.1007/s12553-021-00523-z.

[26] Ouzzani M, Hammady H, Fedorowicz Z, et al. Rayyan—a web and mobile app for systematic reviews. *Syst Rev.* 2016;5(1):210. doi: 10.1186/s13643-016-0384-4.

[27] Veritas Health Innovation. Covidence systematic review software. [Internet]. Available from: www.covidence.org

[28] Enhancing the QUAlity and Transparency Of health Research Network. Your one-stop-shop for writing and publishing high-impact health research. [Internet]. 2023. Available from: https://www.equator-network.org/

[29] Eysenbach G. CONSORT-EHEALTH: improving and standardizing evaluation reports of Web-based and mobile health interventions. *J Med Internet Res.* 2011;13(4):e126. 2012/01/03. doi: 10.2196/jmir.1923.

[30] Manta C, Mahadevan N, Bakker J, et al. EVIDENCE Publication Checklist for Studies Evaluating Connected Sensor Technologies: Explanation and Elaboration. *Digit Biomark.* 2021;5(2):127-147. 2021/06/29. doi: 10.1159/000515835.

[31] Agarwal S, LeFevre AE, Lee J, et al. Guidelines for reporting of health interventions using mobile phones: mobile health (mHealth) evidence reporting and assessment (mERA) checklist. *Bmj.* 2016;352:i1174. 2016/03/19. doi: 10.1136/bmj.i1174.

For peer review only - http://bmjopen.bmj.com/site/about/guidelines.xhtml

[32] Rhon DI, Fritz JM, Kerns RD, et al. TIDieR-telehealth: precision in reporting of telehealth interventions used in clinical trials - unique considerations for the Template for the Intervention Description and Replication (TIDieR) checklist. *BMC Medical Research Methodology.* 2022;22(1):161. doi: 10.1186/s12874-022-01640-7.
[33] Dao KP, De Cocker K, Tong HL, et al. Smartphone-Delivered Ecological Momentary Interventions Based on Ecological Momentary Assessments to Promote Health Behaviors: Systematic Review and Adapted Checklist for Reporting Ecological Momentary Assessment and Intervention Studies. *JMIR Mhealth Uhealth.* 2021;9(11):e22890. 2021/11/23. doi: 10.2196/22890.
[34] Page MJ, McKenzie JE, Bossuyt PM, et al. The PRISMA 2020 statement: an updated guideline for reporting systematic reviews. *BMJ.* 2021;372:n71. doi: 10.1136/bmj.n71.
[35] Grainger R, Devan H, Sangelaji B, et al. Issues in reporting of systematic review methods in health app-focused reviews: A scoping review. *Health Informatics Journal.* 2020;26(4):2930-2945. doi: 10.1177/1460458220952917.
[36] Gasteiger N, Vercell A, Davies A, et al. Patient-facing genetic and genomic mobile apps in the UK: a systematic review of content, functionality, and quality. *Journal of Community Genetics.* 2022;13(2):171-182. doi: 10.1007/s12687-022-00579-y.
[37] Vercell A, Gasteiger N, Yorke J, et al. Patient-facing cancer mobile apps that enable patient reported outcome data to be collected: A systematic review of content, functionality, quality, and ability to integrate with electronic health records. *Int J Med Inform.* 2023;170:104931. 2022/12/04. doi: 10.1016/j.ijmedinf.2022.104931.
[38] Stoyanov S, Hides L, Kavanagh D, et al. Mobile app rating scale: a new tool for assessing the quality of health mobile apps. *JMIR mHealth uHealth.* 2015;3(1):e27. doi: 10.2196/mhealth.3422.
[39] Moher D, Schulz KF, Simera I, et al. Guidance for Developers of Health Research Reporting Guidelines. *PLOS Medicine.* 2010;7(2):e1000217. doi: 10.1371/journal.pmed.1000217.
[40] JBI Scoping Review Methodology Group. JBI manual for evidence synthesis: scoping reviews chapter. [Internet]. 2023. Available from: https://jbi.global/scoping-review-network/about
[41] Arksey H and O'Malley L. Scoping studies: Towards a methodological framework. *International Journal of Social Research Methodology* 2005;8(1):19-32. doi: 10.1080/1364557032000119616.
[42] Tricco AC, Lillie E, Zarin W, et al. PRISMA extension for scoping reviews (PRISMA-ScR): checklist and explanation. *Ann Intern Med.* 2018;169(7):467–473. doi: 10.7326/M18-0850.
[43] Clarke M, Oxman A, Paulsen E, et al. Appendix A: Guide to the contents of a Cochrane Methodology protocol and review. *Cochrane Handbook for Systematic Reviews of Interventions Version 510*. The Cochrane Collaboration, 2011.
[44] Levac D, Colquhoun H and O'Brien KK. Scoping studies: Advancing the methodology. *Implement Sci.* 2010;5(69):1-9. doi: 10.1186/1748-5908-5-69.

For peer review only

For peer review only - http://bmjopen.bmj.com/site/about/guidelines.xhtml

Appendix 1. Preferred Reporting Items for Systematic reviews and Meta-Analyses extension for Scoping Reviews (PRISMA-ScR) Checklist

From: Tricco AC, Lillie E, Zarin W, O'Brien KK, Colquhoun H, Levac D, et al. PRISMA Extension for Scoping Reviews (PRISMAScR): Checklist and Explanation. Ann Intern Med. 2018;169:467–473. doi: 10.7326/M18-0850.

SECTION	ITEM	PRISMA-ScR CHECKLIST ITEM	REPORTED ON PAGE #
**TITLE**			
Title	1	Identify the report as a scoping review.	1
**ABSTRACT**			
Structured summary	2	Provide a structured summary that includes (as applicable): background, objectives, eligibility criteria, sources of evidence, charting methods, results, and conclusions that relate to the review questions and objectives.	3
**INTRODUCTION**			
Rationale	3	Describe the rationale for the review in the context of what is already known. Explain why the review questions/objectives lend themselves to a scoping review approach.	6
Objectives	4	Provide an explicit statement of the questions and objectives being addressed with reference to their key elements (e.g., population or participants, concepts, and context) or other relevant key elements used to conceptualize the review questions and/or objectives.	6
**METHODS**			
Protocol and registration	5	Indicate whether a review protocol exists; state if and where it can be accessed (e.g., a Web address); and if available, provide registration information, including the registration number.	7
Eligibility criteria	6	Specify characteristics of the sources of evidence used as eligibility criteria (e.g., years considered, language, and publication status), and provide a rationale.	8, 9
Information sources	7	Describe all information sources in the search (e.g., databases with dates of coverage and contact with authors to identify additional sources), as well as the date the most recent search was executed.	7
Search	8	Present the full electronic search strategy for at least 1 database, including any limits used, such that it could be repeated.	19
Selection of sources of evidence	9	State the process for selecting sources of evidence (i.e., screening and eligibility) included in the scoping review.	8-10
Data charting process	10	Describe the methods of charting data from the included sources of evidence (e.g., calibrated forms or forms that have been tested by the team before their use, and whether data charting was done independently or in duplicate) and any processes for obtaining and confirming data from investigators.	10
Data items	11	List and define all variables for which data were sought and any assumptions and simplifications made.	10, 11
Critical appraisal of individual sources of evidence	12	If done, provide a rationale for conducting a critical appraisal of included sources of evidence; describe the methods used and how this information was used in any data synthesis (if appropriate).	N/A

For peer review only - http://bmjopen.bmj.com/site/about/guidelines.xhtml

SECTION	ITEM	PRISMA-ScR CHECKLIST ITEM	REPORTED ON PAGE #
Synthesis of results	13	Describe the methods of handling and summarizing the data that were charted.	12
**RESULTS**			
Selection of sources of evidence	14	Give numbers of sources of evidence screened, assessed for eligibility, and included in the review, with reasons for exclusions at each stage, ideally using a flow diagram.	N/A
Characteristics of sources of evidence	15	For each source of evidence, present characteristics for which data were charted and provide the citations.	N/A
Critical appraisal within sources of evidence	16	If done, present data on critical appraisal of included sources of evidence (see item 12).	N/A
Results of individual sources of evidence	17	For each included source of evidence, present the relevant data that were charted that relate to the review questions and objectives.	N/A
Synthesis of results	18	Summarize and/or present the charting results as they relate to the review questions and objectives.	N/A
**DISCUSSION**			
Summary of evidence	19	Summarize the main results (including an overview of concepts, themes, and types of evidence available), link to the review questions and objectives, and consider the relevance to key groups.	N/A
Limitations	20	Discuss the limitations of the scoping review process.	12
Conclusions	21	Provide a general interpretation of the results with respect to the review questions and objectives, as well as potential implications and/or next steps.	N/A
**FUNDING**			
Funding	22	Describe sources of funding for the included sources of evidence, as well as sources of funding for the scoping review. Describe the role of the funders of the scoping review.	13

For peer review only

Appendix 2. Examples of the search strategy.

	SCOPUS	ACM Digital Library (The ACM Guide to Computing Literature)	APA PsycInfo (1806 to January Week 1 2024)	CINAHL Plus (EBSCO)	AMED (Allied and Complementary Medicine) 1985 to October 2023	Embase (1980 to 2024 Week 01)	Ovid MEDLINE(R) ALL (1946 to January 10, 2024)
**Technology**	mobile	mobile	Mobile Phones/	Mobile.mp	mobile.mp.	mobile phone/ or mobile.mp.	mobile.mp.
	smartphone	smartphone	Smartphones/	Smartphone/	Smartphone.mp	smartphone/	Smartphone/
	"cell phone"	"cell phone"	"cell phone".mp	“Cellular Phone”/	"Cell phone".mp	"cell phone".mp.	Cell Phone/
	mhealth	mhealth	mhealth.mp	Mhealth.mp	mhealth.mp.	mhealth.mp.	mhealth.mp.
	"mobile health"	"mobile health"	Mobile Health/	"mobile health".mp.	"mobile health".mp.	"mobile health".mp. or mobile health application/	"mobile health".mp.
	ehealth	ehealth	ehealth.mp.	eHealth.mp.	ehealth.mp.	ehealth.mp. or telehealth/	ehealth.mp.
	tele*	tele*	tele*.mp. Telemedicine/	Telehealth/	Telemedicine/ or tele*.mp.	tele*.mp. or telemedicine/	Telemedicine/ or tele*.mp.
**Review type**	((app OR apps OR application OR applications) W/5 review))	"app* review"	((App or apps or application or applications) adj5 review).mp.	(App or apps or application or applications) N5 review	((app or apps or application or applications) adj5 review).mp.	((app or apps or application or applications) adj5 review).mp.	((app or apps or application or applications) adj5 review).mp.
**Limits**	PUBYEAR > 2006	[E-Publication Date: (01/01/2007 TO 31/12/2024)]	yr="2007 -Current"	Publication Year: 2007-2024; Publication Date: 20070101-20241231	yr="2007 -Current"	yr="2007 -Current"	yr="2007 -Current"

For peer review only - http://bmjopen.bmj.com/site/about/guidelines.xhtml

Appendix 3. PRISMA (2020) checklist.

From: Page MJ, McKenzie JE, Bossuyt PM, Boutron I, Hoffmann TC, Mulrow CD, et al. The PRISMA 2020 statement: an updated guideline for reporting systematic reviews. BMJ 2021;372:n71. doi: 10.1136/bmj.n71

Section and Topic	Item #	Checklist item	Location where item is reported
**TITLE**			
Title	1	Identify the report as a systematic review.	
**ABSTRACT**			
Abstract	2	See the PRISMA 2020 for Abstracts checklist.	
**INTRODUCTION**			
Rationale	3	Describe the rationale for the review in the context of existing knowledge.	
Objectives	4	Provide an explicit statement of the objective(s) or question(s) the review addresses.	
**METHODS**			
Eligibility criteria	5	Specify the inclusion and exclusion criteria for the review and how studies were grouped for the syntheses.	
Information sources	6	Specify all databases, registers, websites, organisations, reference lists and other sources searched or consulted to identify studies. Specify the date when each source was last searched or consulted.	
Search strategy	7	Present the full search strategies for all databases, registers and websites, including any filters and limits used.	
Selection process	8	Specify the methods used to decide whether a study met the inclusion criteria of the review, including how many reviewers screened each record and each report retrieved, whether they worked independently, and if applicable, details of automation tools used in the process.	
Data collection process	9	Specify the methods used to collect data from reports, including how many reviewers collected data from each report, whether they worked independently, any processes for obtaining or confirming data from study investigators, and if applicable, details of automation tools used in the process.	
Data items	10a	List and define all outcomes for which data were sought. Specify whether all results that were compatible with each outcome domain in each study were sought (e.g. for all measures, time points, analyses), and if not, the methods used to decide which results to collect.	
	10b	List and define all other variables for which data were sought (e.g. participant and intervention characteristics, funding sources). Describe any assumptions made about any missing or unclear information.	
Study risk of bias assessment	11	Specify the methods used to assess risk of bias in the included studies, including details of the tool(s) used, how many reviewers assessed each study and whether they worked independently, and if applicable, details of automation tools used in the process.	
Effect measures	12	Specify for each outcome the effect measure(s) (e.g. risk ratio, mean difference) used in the synthesis or presentation of results.	

For peer review only - http://bmjopen.bmj.com/site/about/guidelines.xhtml

Section and Topic	Item #	Checklist item	Location where item is reported
Synthesis methods	13a	Describe the processes used to decide which studies were eligible for each synthesis (e.g. tabulating the study intervention characteristics and comparing against the planned groups for each synthesis (item #5)).	
	13b	Describe any methods required to prepare the data for presentation or synthesis, such as handling of missing summary statistics, or data conversions.	
	13c	Describe any methods used to tabulate or visually display results of individual studies and syntheses.	
	13d	Describe any methods used to synthesize results and provide a rationale for the choice(s). If meta-analysis was performed, describe the model(s), method(s) to identify the presence and extent of statistical heterogeneity, and software package(s) used.	
	13e	Describe any methods used to explore possible causes of heterogeneity among study results (e.g. subgroup analysis, meta-regression).	
	13f	Describe any sensitivity analyses conducted to assess robustness of the synthesized results.	
Reporting bias assessment	14	Describe any methods used to assess risk of bias due to missing results in a synthesis (arising from reporting biases).	
Certainty assessment	15	Describe any methods used to assess certainty (or confidence) in the body of evidence for an outcome.	
**RESULTS**			
Study selection	16a	Describe the results of the search and selection process, from the number of records identified in the search to the number of studies included in the review, ideally using a flow diagram.	
	16b	Cite studies that might appear to meet the inclusion criteria, but which were excluded, and explain why they were excluded.	
Study characteristics	17	Cite each included study and present its characteristics.	
Risk of bias in studies	18	Present assessments of risk of bias for each included study.	
Results of individual studies	19	For all outcomes, present, for each study: (a) summary statistics for each group (where appropriate) and (b) an effect estimate and its precision (e.g. confidence/credible interval), ideally using structured tables or plots.	
Results of syntheses	20a	For each synthesis, briefly summarise the characteristics and risk of bias among contributing studies.	
	20b	Present results of all statistical syntheses conducted. If meta-analysis was done, present for each the summary estimate and its precision (e.g. confidence/credible interval) and measures of statistical heterogeneity. If comparing groups, describe the direction of the effect.	
	20c	Present results of all investigations of possible causes of heterogeneity among study results.	
	20d	Present results of all sensitivity analyses conducted to assess the robustness of the synthesized results.	

For peer review only

Section and Topic	Item #	Checklist item	Location where item is reported
Reporting biases	21	Present assessments of risk of bias due to missing results (arising from reporting biases) for each synthesis assessed.	
Certainty of evidence	22	Present assessments of certainty (or confidence) in the body of evidence for each outcome assessed.	
**DISCUSSION**			
Discussion	23a	Provide a general interpretation of the results in the context of other evidence.	
	23b	Discuss any limitations of the evidence included in the review.	
	23c	Discuss any limitations of the review processes used.	
	23d	Discuss implications of the results for practice, policy, and future research.	
**OTHER INFORMATION**			
Registration and protocol	24a	Provide registration information for the review, including register name and registration number, or state that the review was not registered.	
	24b	Indicate where the review protocol can be accessed, or state that a protocol was not prepared.	
	24c	Describe and explain any amendments to information provided at registration or in the protocol.	
Support	25	Describe sources of financial or non-financial support for the review, and the role of the funders or sponsors in the review.	
Competing interests	26	Declare any competing interests of review authors.	
Availability of data, code and other materials	27	Report which of the following are publicly available and where they can be found: template data collection forms; data extracted from included studies; data used for all analyses; analytic code; any other materials used in the review.	

For peer review only

Appendix 4. PRISMA flowchart for new systematic reviews.

From: Page MJ, McKenzie JE, Bossuyt PM, Boutron I, Hoffmann TC, Mulrow CD, et al. The PRISMA 2020 statement: an updated guideline for reporting systematic reviews. BMJ 2021;372:n71. doi: 10.1136/bmj.n71

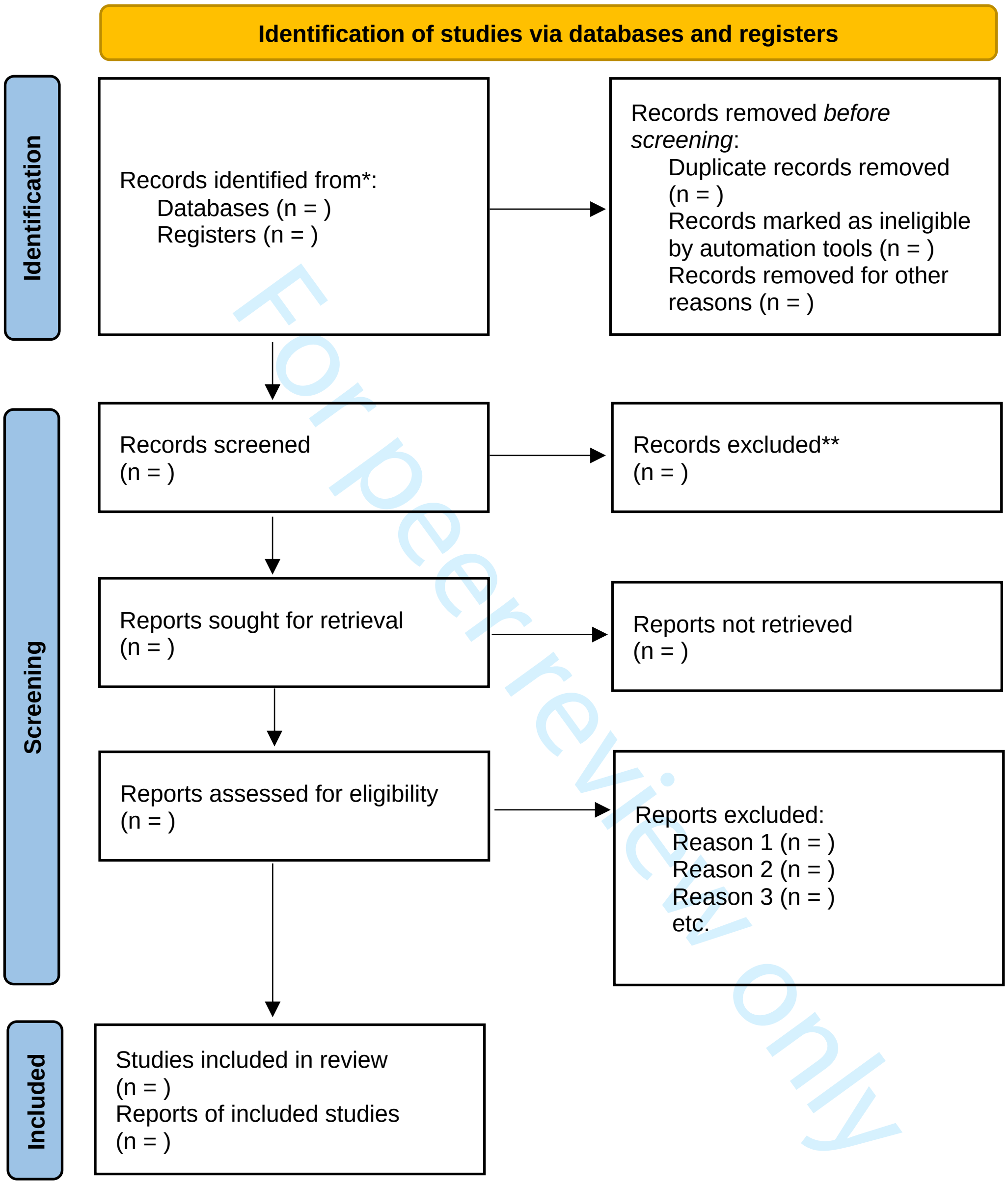

*Consider, if feasible to do so, reporting the number of records identified from each database or register searched (rather than the total number across all databases/registers).

**If automation tools were used, indicate how many records were excluded by a human and how many were excluded by automation tools.

For peer review only

Appendix 5. PRISMA flowchart for updated systematic reviews.

From: Page MJ, McKenzie JE, Bossuyt PM, Boutron I, Hoffmann TC, Mulrow CD, et al. The PRISMA 2020 statement: an updated guideline for reporting systematic reviews. BMJ 2021;372:n71. doi: 10.1136/bmj.n71

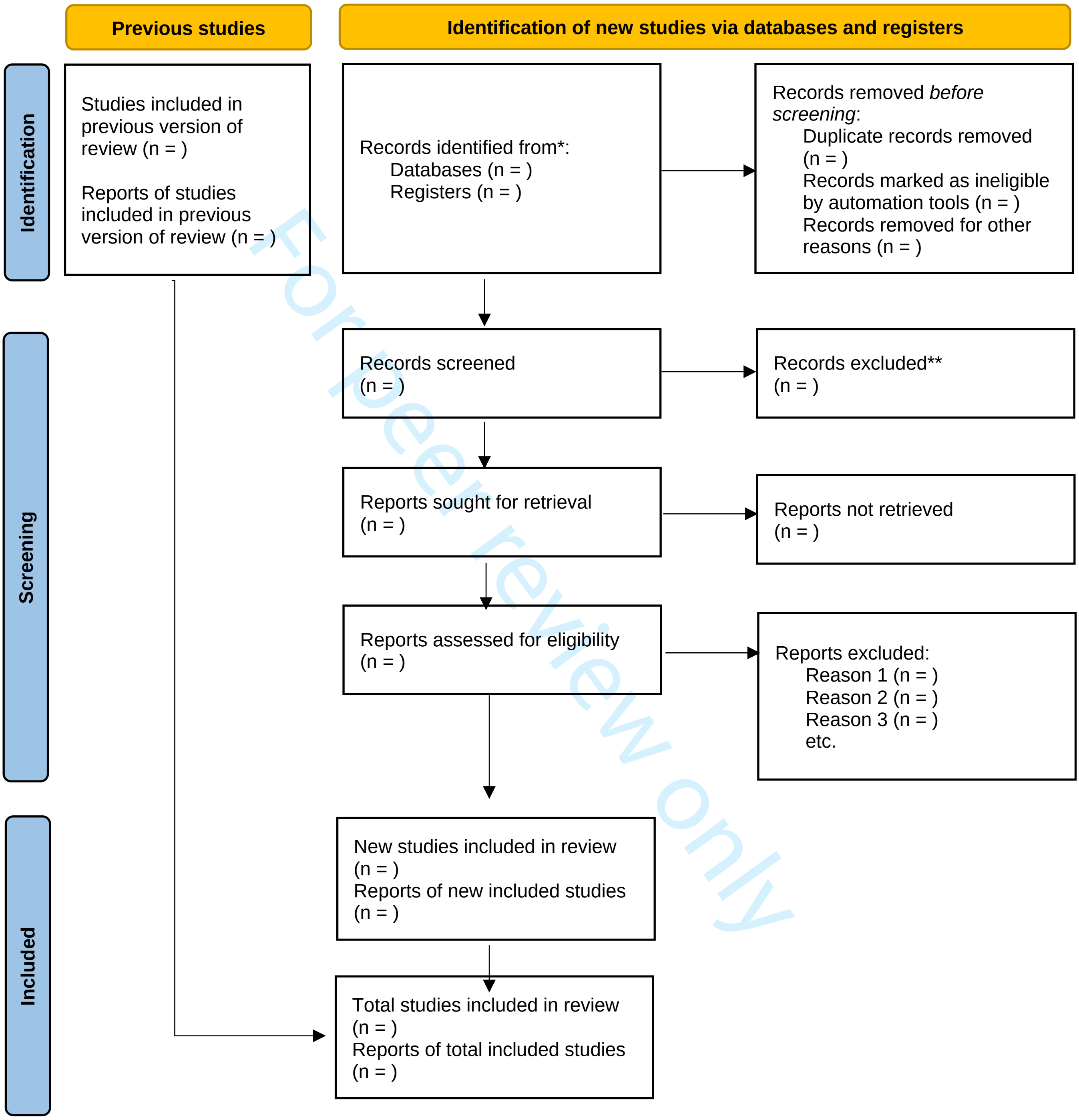

For peer review only

*Consider, if feasible to do so, reporting the number of records identified from each database or register searched (rather than the total number across all databases/registers).

**If automation tools were used, indicate how many records were excluded by a human and how many were excluded by automation tools.

For peer review only - http://bmjopen.bmj.com/site/about/guidelines.xhtml

BMJ Open

The reporting quality of published reviews of commercial and publicly available mobile health apps (mHealth app reviews): A scoping review protocol

Journal:	*BMJ Open*
Manuscript ID	bmjopen-2023-083364.R2
Article Type:	Protocol
Date Submitted by the Author:	17-May-2024
Complete List of Authors:	Gasteiger, Norina; The University of Manchester, Division of Nursing, Midwifery and Social Work; NIHR Applied Research Collaboration Greater Manchester<br>Norman, Gill; The University of Manchester, Division of Nursing, Midwifery & Social Work; NIHR Applied Research Collaboration Greater Manchester<br>Grainger, Rebecca; University of Otago, Department of Medicine<br>Eost-Telling, Charlotte; The University of Manchester, School of Nursing, Midwifery & Social Work; NIHR Applied Research Collaboration Greater Manchester<br>Jones, Debra; The University of Manchester, School of Nursing, Midwifery & Social Work<br>Ali, Syed; The University of Manchester, Centre for Health Informatics, Division of Informatics, Imaging & Data Sciences, Manchester Academic Health Science Centre; NIHR Applied Research Collaboration Greater Manchester<br>van der Veer, Sabine N.; The University of Manchester, Centre for Health Informatics, Division of Informatics, Imaging & Data Sciences, Manchester Academic Health Science Centre; NIHR Applied Research Collaboration Greater Manchester<br>Ford, Claire; The University of Manchester, Division of Nursing, Midwifery & Social Work<br>Hall, Alex; The University of Manchester, Division of Nursing, Midwifery & Social Work<br>Law, Kate; The University of Manchester, Division of Nursing, Midwifery & Social Work; The Christie Hospital NHS Trust<br>Byerly, Matthew; The University of Kansas School of Medicine<br>Davies, Alan; The University of Manchester, Centre for Health Informatics, Division of Informatics, Imaging & Data Sciences, Manchester Academic Health Science Centre<br>Paripoorani, Deborah; Manchester Royal Infirmary, EMERGING Research Team; NIHR Applied Research Collaboration Greater Manchester<br>Shi, Chunhu; The University of Manchester, Division of Nursing, Midwifery & Social Work; NIHR Applied Research Collaboration Greater Manchester<br>Dowding, Dawn; The University of Manchester, Division of Nursing, Midwifery & Social Work; NIHR Applied Research Collaboration Greater Manchester

**Primary Subject Heading**:	Research methods
Secondary Subject Heading:	Health informatics
Keywords:	Information technology < BIOTECHNOLOGY & BIOINFORMATICS, Telemedicine < BIOTECHNOLOGY & BIOINFORMATICS, eHealth, STATISTICS & RESEARCH METHODS

SCHOLARONE™ Manuscripts

The reporting quality of published reviews of commercial and publicly available mobile health apps (mHealth app reviews): A scoping review protocol

Norina Gasteiger[1,2], Gill Norman[1,2,3], Rebecca Grainger[4], Charlotte Eost-Telling[1,2], Debra Jones[1], Syed Mustafa Ali[2,5], Sabine N. van der Veer[2,5], Claire R. Ford[1], Alex Hall[1], Kate Law[1,6], Matthew Byerly[7], Alan Davies[5], Deborah Paripoorani[2,8], Chunhu Shi[1,2] & Dawn Dowding[1,2]

1. Division of Nursing, Midwifery and Social Work, University of Manchester, Manchester, UK
2. National Institute for Health and Care Research Applied Research Collaboration Greater Manchester, Manchester, UK
3. NIHR Innovation Observatory, Population Health Sciences Institute, Newcastle University, Newcastle, UK
4. Department of Medicine, University of Otago Wellington, Wellington, New Zealand
5. Centre for Health Informatics, Division of Informatics, Imaging and Data Sciences, Manchester Academic Health Science Centre, University of Manchester, Manchester, UK
6. The Christie Hospital NHS Foundation Trust, Manchester, UK
7. The University of Kansas School of Medicine-Wichita, Wichita, Kansas, United States of America
8. EMERGING Research Team, Manchester Royal Infirmary, Manchester, UK

For peer review only

AUTHOR DETAILS

	ORCiD	Email address
Dr Norina Gasteiger	0000-0001-7801-7417	norina.gasteiger@manchester.ac.uk
Dr Gill Norman	0000-0002-3972-5733	gill.norman@manchester.ac.uk
Professor Rebecca Grainger	0000-0001-9201-8678	rebecca.grainger@otago.ac.nz
Dr Charlotte Eost-Telling	0000-0002-9568-3195	charlotte.eost-telling@manchester.ac.uk
Mrs Debra Jones	0000-0003-1479-3622	debra.jones@manchester.ac.uk
Mr Syed Mustafa Ali	0000-0001-9393-9049	syedmustafa.ali@manchester.ac.uk
Dr Sabine N. van der Veer	0000-0003-0929-436X	sabine.vanderveer@manchester.ac.uk
Dr Claire R. Ford	0000-0003-2950-1565	claire.ford@manchester.ac.uk
Dr Alex Hall	0000-0002-8849-744X	alex.hall@manchester.ac.uk
Ms Kate Law	0000-0002-0995-8309	kate.law@postgrad.manchester.ac.uk
Professor Matthew Byerly	0000-0001-7033-1268	mbyerly3@kumc.edu
Dr Alan Davies	0000-0001-5737-5629	alan.davies-2@manchester.ac.uk
Ms Deborah Paripoorani	N/A	Deborah.Paripoorani@mft.nhs.uk
Dr Chunhu Shi	0000-0003-0151-0451	chunhu.shi@manchester.ac.uk
Professor Dawn Dowding	0000-0001-5672-8605	dawn.dowding@manchester.ac.uk

For peer review only

For peer review only - http://bmjopen.bmj.com/site/about/guidelines.xhtml

ABSTRACT

Introduction

Reviews of commercial and publicly available smartphone (mobile) health applications (mHealth app reviews) are being undertaken and published. However, there is variation in the conduct and reporting of mHealth app reviews, with no existing reporting guidelines. Building on the PRISMA guidelines, we aim to develop the Consensus for APP Review Reporting Items (CAPPRRI) guidance, to support the conduct and reporting of mHealth app reviews. This scoping review of published mHealth app reviews will explore their alignment, deviation, and modification to the PRISMA 2020 items for systematic reviews and identify a list of possible items to include in CAPPRRI.

Method and analysis

We are following the Joanna Briggs Institute approach and Arksey and O'Malley's five-step process. Patient and public contributors, mHealth app review, digital health research and evidence synthesis experts, healthcare professionals and a specialist librarian gave feedback on the methods. We will search SCOPUS, CINAHL Plus, AMED, EMBASE, Medline, APA PsycINFO and ACM Digital Library for articles reporting mHealth app reviews and use a two-step screening process to identify eligible articles. Information on whether the authors have reported, or how they have modified the PRISMA 2020 items in their reporting will be extracted. Data extraction will also include the article characteristics, protocol and registration information, review question frameworks used, information about the search and screening process, how apps have been evaluated, and evidence of stakeholder engagement. This will be analysed using a content synthesis approach and presented using descriptive statistics and summaries.

Ethics and dissemination

Ethical approval is not required. The findings will be disseminated through peer-reviewed journal publications (shared on our project website and on the EQUATOR Network website where the CAPPRRI guidance has been registered as under development), conference presentations and blog and social media posts in lay language.

Registration

This protocol is registered on OSF (https://osf.io/5ahjx).

For peer review only

KEYWORDS

app review; mHealth; smartphone; research methods; reporting; scoping review

STRENGTHS AND LIMITATIONS

- This review will be conducted systematically, with data extraction informed by the PRISMA 2020 reporting items and a previous scoping review.
- The protocol has had input from a multidisciplinary team of mHealth app review, digital health and evidence synthesis experts, healthcare professionals, a librarian, and patient and public contributors.
- The broad scope of health topics to be included in the review increases the generalisability of the findings.
- In line with scoping review guidance, a quality appraisal of the included studies will not be conducted.
- Only mHealth app reviews reported in English will be included, meaning some other relevant reviews published in other languages may not be included.

WORD COUNT: 2,789

For peer review only

INTRODUCTION

In 2021 it was recorded that there are more than 350,000 health applications (apps) (1). These applications are increasingly being integrated into healthcare; supporting professionals in their clinical practice (2) and empowering patients to manage and monitor their health conditions (3, 4). However, the quality and reliability of mobile health (mHealth) apps varies significantly (5), as developers can release smartphone health apps without any evaluation, meaning it is a challenge for health professionals and users without expertise in health research and digital technology to identify and evaluate the suitability of mHealth apps for their use.

This has led to the emergence of a new method: systematic reviews of commercial and publicly available mHealth apps (hereafter called mHealth app reviews). This provides a standard approach to identify mHealth apps relevant to a particular use case and assess aspects such as quality and functionality. Many mHealth app reviews have now been published, for varied topics including genetics (6, 7), patient-reported outcomes in oncology (8), mental health (9, 10), rheumatoid arthritis (11), strength training (12), menopause (13), exercise (14, 15), hand hygiene (16), atrial fibrillation (17), pain (18-20) and smoking cessation (21, 22). These reviews can serve as a valuable resource for healthcare decision-makers, practitioners, patients and the general public seeking high-quality mHealth apps; can identify gaps in the field and may guide researchers and industry in developing new products.

While mHealth app reviews share features with traditional systematic literature reviews, they differ substantially in their methods and reporting (23) due to the review being of commercial and publicly available products on app stores, instead of published literature. Examples of traditional systematic reviews of literature describing apps include those on monitoring and managing mental health symptoms (24) and self-managing pregnancy (25). While literature reviews can tell us about the effectiveness of apps which have been evaluated and the results published, they do not provide a comprehensive overview of all apps that are commercially or publicly available for use by patients, healthcare professionals and the public. mHealth app reviews also differ as there are no formal requirements for the protocol to be registered, searches take place on app libraries, screening often takes place on Excel (rather than using specifically designed tools like Rayyan (26) or Covidence (27)), and they are more challenging to replicate, as apps may emerge, disappear or be updated between searches (23).

The EQUATOR Network (28) provides an array of guidelines for reporting evaluations of digital technologies, such as the CONSORT-EHEALTH checklist (an extension of the CONSORT checklist tailored for reporting randomised controlled trials of web-based and mobile health interventions (29)), and guidance on reporting evaluations of specific technologies, such as sensors (30), mHealth interventions (31), telehealth in clinical trials (32) and smartphone-delivered ecological momentary assessments (33). There are also several extensions of the Preferred Reporting Items for Systematic Reviews and Meta-Analyses (PRISMA) reporting guidelines (34) available for different types of literature reviews.

In contrast, no reporting guidelines exist for mHealth app reviews, and we are not aware of any currently in development. The need for a reporting standard for health app-focused reviews was emphasised in a scoping review published in 2020 (35). The authors reviewed 26 mHealth app reviews published between 2012 and 2018 and found issues in their reporting. For example, the date of the searches was sometimes unclear (38%, 10/26) or absent (15%, 4/26). The number of reviewers

For peer review only - http://bmjopen.bmj.com/site/about/guidelines.xhtml

involved was also poorly reported in 58% of reviews, and in 83% it was unclear whether screening was performed independently. Another important finding was the absence of clinical recommendations or reporting on clinical efficacy, found in 77% of the included reviews. Adhering to reporting guidelines may help to minimise the presence of these inconsistencies in reporting, and ensure that standardised information is presented.

If a key purpose of an mHealth app review is for people to be able to identify the best product for a particular purpose, it is important to further explore how app review authors have evaluated the apps and reported on their outcomes. For example, some previous app reviews have considered the accessibility of the apps by generating readability metrics on the written content (13, 36, 37). This is especially important for patient or public-facing apps. There are also various approaches to reporting on efficacy (i.e., that the app results in intended outcomes). This may include searching for evidence of a previous evaluation within the app itself, on developers' websites, or for published literature or conference abstracts in academic databases. For example the Mobile App Rating Scale (MARS) (38) is commonly used in mHealth app reviews and has an item which addresses *whether an app has been trialled or tested.* Previous examples of app reviews have approached this item by excluding the item entirely (11) or searching Google Scholar (6, 8, 13, 16, 18). It is unclear how other authors have evaluated the apps and how this has informed their recommendations of apps as being high-quality. Understanding the nature of the evaluations (especially efficacy evidence) seems essential in mHealth app reviews as readers, including health and care workers, patients and the public, and healthcare decision-makers may use these to choose which apps to use.

We have previously discussed the methodological considerations for conducting systematic mHealth app reviews; introducing a 7-step method and the TECH framework (Target user, Evaluation focus, Connectedness and the Health domain) for developing research (review) questions and determining app eligibility criteria (23). This is the first stage of a broader project that aims to systematise the process of conducting and reporting mHealth app reviews.

The next step is to develop reporting guidelines to support authors of mHealth app reviews in transparently presenting their methods and findings. The field of app reviews is rapidly developing and expanding, so a new scoping review is required to update that previously reported, which had a search date of 2018 (35). A preliminary search of the Cochrane Database of Systematic Reviews, Google Scholar and JBI Evidence Synthesis was conducted, and no current or ongoing systematic reviews or scoping reviews on the topic were identified. Therefore, we are undertaking a scoping review to build on and update the scoping review by Grainger et al. (35).

OBJECTIVES

In line with guidance for developing reporting guidelines (39), the next step is to search for relevant evidence on the quality of the reporting of published mHealth app reviews. The aim of this work is, therefore, to conduct a scoping review on published mHealth app reviews to explore their alignment, deviation and modification to the PRISMA 2020 items and identify a list of possible items to include in the new Consensus for APP Review Reporting Items (CAPPRRI) guideline.

METHODS

Scoping review

For peer review only - http://bmjopen.bmj.com/site/about/guidelines.xhtml

The methods for this scoping review were developed in alignment with the Joanna Briggs Institute approach for scoping reviews (40) and reviewed by a group of patient and public contributors and an advisory group consisting of mHealth app review, digital health research and evidence synthesis experts and NHS healthcare professionals interested in app reviews. The review will be carried out using the five-step process for conducting scoping reviews, originally outlined by Arksey and O'Malley (41). This protocol has already been registered and made publicly available on OSF (https://osf.io/5ahjx).The final review will be reported using the PRISMA-ScR extension guidelines for scoping reviews (42). This protocol has been reported using the PRISMA-P extension (43) (see Appendix 1). We will start the review on the 2nd January 2024 and complete it by the 2nd September, 2024.

Procedures

Identifying the initial research question

We used the Study, Data, Methods and Outcomes (SDMO) acronym to inform our research questions and eligibility criteria, which has been recommended when conducting reviews on methodology or theory (44). Additionally, as suggested by Levac et al. (45), we considered the purpose and expected outputs of the review to assist in writing the research questions. The purpose and expected outputs are primarily a list of potential reporting items used to inform the future CAPPRRI guideline. Building on the PRISMA 2020 (34) items is appropriate as many app review authors already informally use the PRISMA items to report their work or have attempted to amend the PRISMA flowchart when reporting their app search and screening process (6, 8, 11-13, 16-18, 21).

The second question seeks to understand what outcomes were evaluated in mHealth app reviews (e.g., usability, functionality, privacy, accessibility and efficacy). This builds on the previous review (35), which found that most of the app reviews did not make clinical recommendations or report clinical efficacy (i.e., whether the app could meet desired outcomes in a clinical context). We are therefore interested in understanding what the outcomes were in general, and whether any of the app reviews reported on efficacy in any other sense such as satisfaction, increased knowledge or perceived support.

The two key questions are:

1. In published reviews of commercial and publicly available mHealth apps how does reporting align with or deviate from the PRISMA 2020 items? Have authors used items that directly align with PRISMA 2020 items or have these been modified?
2. What outcomes did the mHealth app reviews evaluate and report on?

Identifying relevant studies

We will search the SCOPUS, CINAHL Plus (via EBSCO), AMED (via Ovid), EMBASE (via Ovid), Medline (via Ovid), APA PsycINFO and ACM Digital Library databases for published mHealth app reviews, under the guidance of a teaching and learning librarian who has given input on the search strategy. Reference lists of eligible articles will also be hand-searched for additional sources (snowballing), while a forward citation approach will be used to identify app reviews that have cited earlier published work.

For peer review only

The key terms used to build the search strategy are shown in Table 1, with the full search strategy presented in Appendix 2. Where appropriate, subject headings will be applied in the databases. The keywords will be separated by the ‘OR’ Boolean operator. The technology and review type keywords will be separated by the ‘AND’ Boolean operator. The proximity function using five words will be used for the review type keywords, to include different variations of app review, such as review of apps, review of smartphone apps, or review of patient-facing mobile health applications.

Publication date will be limited from 1st January 2007, as the first iPhone (and first smartphone) was introduced on 29th June 2007, so there will be no apps available to review before 2007.

Table 1. Keywords used to identify literature.

**Technology**	**Review type**
• Mobile<br>• Smartphone<br>• “Cell phone”<br>• mHealth<br>• “mobile health”<br>• eHealth<br>• Tele* (to include telehealth, telemedicine and other variations)	(App or apps or application or applications) adj5[1] review

Note:
[1]The exact wording depends on the database and may include adj5, W/5 or N5. The ACM Digital Library does not allow the proximity function, so “app* review” will be used instead.

Study selection

Table 2 presents the eligibility criteria for the literature, using the SDMO acronym (44). Broadly, our inclusion criteria are:

- Reviews of commercial and publicly available mobile (smartphone) apps published in English and after 1 January 2007 (types of study)
- that have a health focus (type of data),
- include any method and measure of evaluating apps (e.g., MARS or user ratings) and,
- have any outcome or focus, including focussing on the availability of apps or evaluation (e.g., quality, functionality, privacy and security or adherence to clinical guidelines).

We will only include mHealth app reviews published in English. However, we will separately list articles that were excluded due to language, which can enable others to easily identify these papers for subsequent reviews.

For peer review only

Table 2. Eligibility criteria for the mHealth app reviews to be included in the scoping review.

**SDMO Acronym**	**Inclusion criteria**	**Exclusion criteria**	**No limits**
Types of study	Reviews of commercial and publicly available mobile apps<br>• *Must be focussed on smartphone (mobile) apps.*<br>• *Can be identified as systematic, scoping or without a specific approach named.*<br>• *Some app reviews may be combined with other literature reviews or reviews of other apps. These will only be included if detail is reported separately on the smartphone app review methods and results.*<br>• *Reviews including other technology (e.g., iPads, digital assistants, virtual reality headsets or smartwatches) will only be included if the focus is on smartphone apps and the other technology is used only to operationalise some of the functions.*<br><br>English language<br><br>Published on or after 1 January, 2007	Literature reviews<br><br>Reviews of other technology or apps (e.g., websites, computer apps, iPad apps)<br><br>Full-text not available<br>• *Exclude abstracts and documents where there is insufficient information or the full-text is not available.*<br><br>Not in English<br><br>Published before 1 January, 2007	Document type<br>• *Any document type will be included, if there is a full-text available so that enough information can be extracted (e.g., full-length conference papers, journal articles, book chapters).*<br><br>Smartphone device<br><br>Operating system requirements<br><br>App markets<br><br>Geography (location)
Types of data	Health focus<br><br>*Must be focussed on a health topic, whereby the apps are marketed for physical or mental health or general well-being. This may include (but is not limited to) apps that educate, empower or inform users on a health topic (e.g., genetics), self-monitor/manage or change health behaviours (e.g., sleep, nutrition, exercise or smoking cessation) or are used for social support or in health systems or by patients, administrators and health and care workers or decision-makers (e.g., screening, diagnosis, triage, appointment-booking, remote monitoring, decision-making, training and treatment).*	Not focussed on health topics	Health topic<br>• *Apps can be for any health topic.*<br><br>Intended users of apps<br>• *Apps can be for any stakeholder, including patients, the public, health professionals and the health system.*
Types of methods	Any method (and measure) of evaluating apps can be included, such as using validated measures (e.g., MARS (38)), synthesising content presented within the app or user ratings and reviews on app markets.	N/A	App evaluation measures and methods.
Types of outcomes	Any outcome, including those related to evaluating app quality, functionality, privacy and security, accessibility or efficacy. This would also include app reviews that simply focus on identifying which apps were available, summarised their content or described the extent to which they adhere to best clinical practice/guidelines.	N/A	Any outcomes.

For peer review only

For peer review only - http://bmjopen.bmj.com/site/about/guidelines.xhtml

The final search results will be imported into Rayyan (26) for deduplication and screening. As suggested by Levac et al. (45), the screening process will be iterative and use a team-based approach. An initial meeting will be held with all researchers involved in the screening process to discuss interpretation of the eligibility criteria and reach shared understanding after an initial set of records have been pilot screened. A two-step process will then be followed. First, two researchers will independently review each abstract/title against the eligibility criteria. Second, the full text of records potentially eligible based on abstract/title screening will be reviewed by two researchers independently. A meeting will be held after the second stage to discuss and reach consensus where there is disagreement. A third reviewer will make the final decision if consensus cannot be reached. Depending on the number of articles, two teams of two researchers may perform the screening process, with a fifth researcher available to resolve disagreements. The search and screening process will be presented as a flowchart.

Charting the data

A data extraction sheet will be created in Excel using headers related to our 7-step method and TECH framework (23), the PRISMA 2020 items (34), whether and how authors modified them, additional information reported, and methods used to appraise the apps' quality, functionality or efficacy. Some items have also been taken from the review conducted by Grainger et al. (35) as these capture details unique to app reviews (e.g., how app stores were searched). Table 3 presents the data extraction items.

Table 3. Information to be extracted from the articles.

**Article characteristics**	
Title	• Title of the article.<br>• Does it name a review method in the title (e.g., systematic, scoping, app review)?
Date	• Year of publication.
Journal	• Name of the journal the app review is published in.<br>• Name and contact information of the editor-in-chief.[1]
Authors	• Name and contact information of all authors.[1]<br>• Source of funding (if any).
Objective	• Aim or research question.
Topic and context	• The health problem being explored.<br>• App target user.<br>• Context: where the app is to be used, including location of care (acute, primary health care, community, long-term care, etc.) and geographical location.
**Protocol and registration**	
A priori review registration	• Was the study protocol registered and was the protocol available?[2]
**Review question**	
Review question frameworks	• Was a framework used to write the review question (e.g., PICO)? If so, which?<br>• Did this align with any of the TECH components? If so, which?
**Reporting guidelines**	
Reporting guidelines	• Did authors state which reporting guideline they used? If so, which?<br>• Did the authors clearly mention amending any guidelines?
Alignment, deviation and modification to the PRISMA (2020) items (see Appendix 3)	• For each of the PRISMA (2020) items, identify whether the information was reported as is, or how this was modified (if applicable).
**Search and screening**	

For peer review only - http://bmjopen.bmj.com/site/about/guidelines.xhtml

Flow charts/diagrams	• Did the authors present a flow diagram for the search and screening process?<br>• Did the authors report a PRISMA (2020) flow chart for new or updated systematic reviews (see Appendix 4 and 5)?<br>• Were amendments made to the PRISMA (2020) flow charts (see Appendix 4 and 5)? If yes, which?
App store search	• Were the apps store(s) searched described including the a) keywords, b) countries/location and c) a clear description of dates of the search?[2]<br>• Was the method for identifying and removing duplicate apps clearly described, including metadata (e.g., version numbers) used to determine if apps were duplicates?[2]<br>• Were the number and independence of people screening apps described?[2]<br>• Were limits on inclusion of apps based on other factors clearly described (lite or full version, paid or free versions, non-English)?[2]<br>• Where the same app featured in different app stores (multiplatform apps), was there a clear statement of which apps were included?[2]<br>• When apps were downloaded to phones for data extraction, were the phone model and version of the operating system clearly reported?[2]
**Evaluating the apps and making recommendations**	
Outcomes	• Which outcomes did the review evaluate? e.g., quality, functionality, usability, privacy, efficacy, accessibility or other
Quality, functionality, usability and other assessments	• Was a best practice content tool (e.g., clinical guideline) used to evaluate app content or quality? Was the source identified, were any modifications made described, and was the use of the guideline justified?[2]<br>• Was an app quality, functionality and/or usability evaluation undertaken using established measures and frameworks (e.g., MARS)?<br>• Are any other tools/instruments used to evaluate app quality and/or usability, and was the source of these described?[2]<br>• Were security and privacy considered? If so, what aspects were included? e.g., login, password, privacy policy, access to microphone or camera, encryption or data sharing.
Efficacy	• Did the authors report on the efficacy of the apps?<br>• If yes, where did they obtain this information and what methods were used?
Accessibility	• Was the accessibility of the apps evaluated, considering different needs of target users?
Recommendations	• Were any apps recommended for use in the clinical setting or by people with chronic health conditions?[2]<br>• Were any apps recommended overall? What informed this recommendation?
**Stakeholder engagement or consultation**	
Patient, public and expert engagement	• Were any patients or members of the public involved in the app review?<br>• Were any other stakeholders involved or consulted (e.g., industry partners, clinicians or software developers)? If so, which?<br>• Was a lay summary provided? If so, were any apps clearly recommended?
**Other**	
Other domains	• Any other domains reported on? If yes, what and how?

Notes:

[1] This information will not be published publicly as part of the review. It will be used to create a database of authors of mHealth app reviews and journals who have published them. This is required for the next step of the

For peer review only - http://bmjopen.bmj.com/site/about/guidelines.xhtml

project- the Delphi (consensus-building) study, as mHealth experts will be participants and potential partners in this process.

[2] These data extraction items were taken from the scoping review conducted by Grainger et al. (35).

Consistent with recommendations by Levac et al. (45), we will take an iterative approach to charting by continually updating the data-charting form as needed. The research team will first pilot the data extraction sheet, by extracting the data from one app review, with discussion afterward to ensure consistency in interpretation of the items. Data will then be extracted from the other articles, with one author extracting the information and another checking this. Depending on the final number of included articles, this will be split between the researchers.

It has been suggested that some scoping reviews should also include quality assessments of the methodology used in the articles (45). However, as this is not the focus of our review and as no specific quality assessment tool currently exists for mHealth app reviews, the quality of the included studies will not be assessed.

Collating, summarising, and reporting the results

Similar to the previous review (35) and as recommended by Arksey and O'Malley (41), we will report data as frequencies (where possible) to determine which items were reported as is, or whether they were modified.

Information that cannot be reported as frequencies, on how the PRISMA 2020 items were modified and how other relevant information was reported will be summarised using a content synthesis approach, to help inform amendments to the existing guideline and to identify new items.

The results overall will be reported using descriptions and examples, while some of the numerical results will also be presented using tables and figures.

Strengths and limitations

This scoping review will be conducted in a systematic and rigorous manner, with data extraction informed by the existing PRISMA 2020 (34) reporting items and a previous scoping review (35). It also adheres to existing guidance on conducting scoping reviews, including from the Joanna Briggs Institute (40) and Arksey and O'Malley (41), and has had input from a multidisciplinary team of mHealth app review, digital health and evidence synthesis experts, NHS healthcare professionals, a librarian and patient and public contributors. The breadth of its scope of health topics (and methodological focus), also means that the findings will be widely generalisable.

A fundamental limitation is the inability to assess the quality of the included reviews, due to an absence of quality appraisal tools for reviews of commercial and publicly available mHealth apps. This limitation means that low quality app reviews may also contribute to the development of the future CAPPRRI guideline. However, we will mitigate this in the next phase of the project, in which a Delphi study with experts will help to prioritise the items. Another limitation concerns restricting the included mHealth app reviews to those reported in English which may lead to other relevant reviews being excluded.

For peer review only

Patient and public involvement

We have established a PPIE group to give feedback on all aspects of our project. The group has been consulted to provide input on the protocol and suggested additional items that should be extracted (i.e., whether the accessibility of the apps was evaluated and whether a lay summary was provided). They also gave ideas for how the findings should be disseminated. We will continue to consult with them throughout the review; this will inform the iterative aspects of the scoping review process and will help to guide the findings and their dissemination.

ETHICS AND DISSEMINATION

Ethical approval is not required to conduct this scoping review which will use only previously published data.

The findings of this scoping review will be disseminated through peer-reviewed journal publications which will be shared on our project website and on the EQUATOR Network website where the CAPPRRI guideline has been registered as under development, in addition to conference presentations and blog posts and short summaries in lay language on professional social media.

CONCLUSION

This protocol describes how we will conduct a scoping review on published mHealth app reviews to explore their alignment, deviation and modification to the PRISMA 2020 items and identify a list of possible items to include in the new CAPPRRI reporting guideline. The results will inform the next phase in developing the CAPPRRI guideline: a Delphi study to reach a consensus on which items are most relevant and important to include in the guideline.

DECLARATIONS

Author contributions

NG, GN and DD designed the protocol. NG wrote the first draft of the manuscript, with support from GN. NG, GN, RG, CE-T, DJ, SMA, SNvdV, CRF, AH, KL, MB, AD, DP, CS and DD revised the protocol critically and approved the final manuscript.

Funding

This work is funded by the National Institute for Health and Care Research Applied Research Collaboration Greater Manchester (Grant award: NIHR200174). The views expressed in this publication are those of the authors and not necessarily those of the National Institute for Health and Care Research or the Department of Health and Social Care.

Acknowledgements

We would like to acknowledge Mr Michael Stevenson for assisting with the search strategy. We would also like to thank our patient and public contributors for their input- thank you Amber McAvoy, Eric Lowndes, Ashgan Mahyoub and Beatrice Namu. We acknowledge Mrs Amy Vercell and Dr Lisa McGarrigle who are part of our team.

For peer review only - http://bmjopen.bmj.com/site/about/guidelines.xhtml

Competing interests

The authors have no competing interests to declare.

Patient consent for publication

Not required.

Data availability

No new data has been generated for this protocol.

References

[1] IQVIA. Digital Health Trends 2021: Innovation, evidence, regulation and adoption. [Internet]. 2021. Available from: https://www.iqvia.com/insights/the-iqvia-institute/reports-and-publications/reports/digital-health-trends-2021

[2] Mayer MA, Rodríguez Blanco O and Torrejon A. Use of Health Apps by Nurses for Professional Purposes: Web-Based Survey Study. *JMIR Mhealth Uhealth.* 2019;7(11):e15195. 2019/11/05. doi: 10.2196/15195.

[3] Dsouza SM, Shetty S, Venne J, et al. Effectiveness of self-management applications in improving clinical health outcomes and adherence among diabetic individuals in low and middle-income countries: a systematic review. *BMJ Open.* 2022;12(11):e060108. 2022/11/16. doi: 10.1136/bmjopen-2021-060108.

[4] Doumen M, De Cock D, Van Lierde C, et al. Engagement and attrition with eHealth tools for remote monitoring in chronic arthritis: a systematic review and meta-analysis. *RMD Open.* 2022;8(2) 2022/10/28. doi: 10.1136/rmdopen-2022-002625.

[5] Larsen ME, Huckvale K, Nicholas J, et al. Using science to sell apps: Evaluation of mental health app store quality claims. *NPJ Digit Med.* 2019;2:18. 2019/07/16. doi: 10.1038/s41746-019-0093-1.

[6] Gasteiger N, Vercell A, Davies A, et al. Patient-facing genetic and genomic mobile apps in the UK: a systematic review of content, functionality, and quality. *J Community Genet.* 2022;13(2):171-182. 2022/02/20. doi: 10.1007/s12687-022-00579-y.

[7] Talwar D, Yeh Y, Chen W-J, et al. Characteristics and quality of genetics and genomics mobile apps: a systematic review. *Eur J Hum Genet.* 2019;27:833–840. doi: https://doi.org/10.1038/s41431-019-0360-2.

[8] Vercell A, Gasteiger N, Yorke J, et al. Patient-facing cancer mobile apps that enable patient reported outcome data to be collected: A systematic review of content, functionality, quality, and ability to integrate with electronic health records. *Int J Med Inform.* 2022;170:104931. 2022/12/04. doi: 10.1016/j.ijmedinf.2022.104931.

[9] Martín-Martín J, Muro-Culebras A, Roldán-Jiménez C, et al. Evaluation of Android and Apple Store Depression Applications Based on Mobile Application Rating Scale. *Int J Environ Res Public Health.* 2021;18(23) 2021/12/11. doi: 10.3390/ijerph182312505.

[10] Tsai Z, Kiss A, Nadeem S, et al. Evaluating the effectiveness and quality of mobile applications for perinatal depression and anxiety: A systematic review and meta-analysis. *Journal of Affective Disorders.* 2022;296:443-453. doi: https://doi.org/10.1016/j.jad.2021.09.106.

[11] Grainger R, Townsley H, White B, et al. Apps for People With Rheumatoid Arthritis to Monitor Their Disease Activity: A Review of Apps for Best Practice and Quality. *JMIR Mhealth Uhealth.* 2017;5(2):e7. doi: 10.2196/mhealth.6956.

For peer review only

[12] McGarrigle L, Boulton E and Todd C. Map the apps: a rapid review of digital approaches to support the engagement of older adults in strength and balance exercises. *BMC Geriatr.* 2020;20(1):483. 2020/11/20. doi: 10.1186/s12877-020-01880-6.
[13] Paripoorani D, Gasteiger N, Hawley-Hague H, et al. A systematic review of menopause apps with an emphasis on osteoporosis. *BMC Womens Health.* 2023;23(1):518. 2023/09/29. doi: 10.1186/s12905-023-02612-9.
[14] Knight E, Stuckey MI, Prapavessis H, et al. Public Health Guidelines for Physical Activity: Is There an App for That? A Review of Android and Apple App Stores. *JMIR mHealth uHealth.* 2015;3(2):e43. doi: 10.2196/mhealth.4003.
[15] Payne HE, Moxley VB and MacDonald E. Health Behavior Theory in Physical Activity Game Apps: A Content Analysis. *JMIR Serious Games.* 2015;3(2):e4. 2015/07/15. doi: 10.2196/games.4187.
[16] Gasteiger N, Dowding D, Ali SM, et al. Sticky apps, not sticky hands: A systematic review and content synthesis of hand hygiene mobile apps. *J Am Med Inform Assoc.* 2021;28(9):2027-2038. 2021/06/29. doi: 10.1093/jamia/ocab094.
[17] Pearsons A, Hanson CL, Gallagher R, et al. Atrial fibrillation self-management: a mobile telephone app scoping review and content analysis. *Eur J Cardiovasc Nurs.* 2021;20(4):305-314. 2021/02/24. doi: 10.1093/eurjcn/zvaa014.
[18] Ali SM, Lau WJ, McBeth J, et al. Digital manikins to self-report pain on a smartphone: A systematic review of mobile apps. *Eur J Pain.* 2021;25(2):327-338. 2020/10/29. doi: 10.1002/ejp.1688.
[19] Kheirinejad S, Visuri A, Suryanarayana SA, et al. Exploring mHealth applications for self-management of chronic low back pain: A survey of features and benefits. *Heliyon.* 2023;9(6):e16586. 2023/06/22. doi: 10.1016/j.heliyon.2023.e16586.
[20] Devan H, Farmery D, Peebles L, et al. Evaluation of Self-Management Support Functions in Apps for People With Persistent Pain: Systematic Review. *JMIR Mhealth Uhealth.* 2019;7(2):e13080. 2019/02/13. doi: 10.2196/13080.
[21] Seo S, Cho SI, Yoon W, et al. Classification of Smoking Cessation Apps: Quality Review and Content Analysis. *JMIR Mhealth Uhealth.* 2022;10(2):e17268. 2022/02/18. doi: 10.2196/17268.
[22] Chen J, Chu J, Marsh S, et al. Smartphone App-Based Interventions to Support Smoking Cessation in Smokers with Mental Health Conditions: A Systematic Review. *Psych* 5: 1077-1100. DOI: 10.3390/psych5040072.
[23] Gasteiger N, Dowding D, Norman G, et al. Conducting a systematic review and evaluation of commercially available mobile applications (apps) on a health-related topic: the TECH approach and a step-by-step methodological guide. *BMJ Open.* 2023;13(e073283). doi: 10.1136/bmjopen-2023-073283.
[24] Wang K, Varma DS and Prosperi M. A systematic review of the effectiveness of mobile apps for monitoring and management of mental health symptoms or disorders. *Journal of Psychiatric Research.* 2018;107:73-78. doi: https://doi.org/10.1016/j.jpsychires.2018.10.006.
[25] Iyawa GE, Dansharif AR and Khan A. Mobile apps for self-management in pregnancy: a systematic review. *Health and Technology.* 2021;11(2):283-294. doi: 10.1007/s12553-021-00523-z.
[26] Ouzzani M, Hammady H, Fedorowicz Z, et al. Rayyan—a web and mobile app for systematic reviews. *Syst Rev.* 2016;5(1):210. doi: 10.1186/s13643-016-0384-4.
[27] Veritas Health Innovation. Covidence systematic review software. [Internet]. Available from: www.covidence.org
[28] Enhancing the QUAlity and Transparency Of health Research Network. Your one-stop-shop for writing and publishing high-impact health research. [Internet]. 2023. Available from: https://www.equator-network.org/
[29] Eysenbach G. CONSORT-EHEALTH: improving and standardizing evaluation reports of Web-based and mobile health interventions. *J Med Internet Res.* 2011;13(4):e126. 2012/01/03. doi: 10.2196/jmir.1923.

For peer review only - http://bmjopen.bmj.com/site/about/guidelines.xhtml

[30] Manta C, Mahadevan N, Bakker J, et al. EVIDENCE Publication Checklist for Studies Evaluating Connected Sensor Technologies: Explanation and Elaboration. *Digit Biomark.* 2021;5(2):127-147. 2021/06/29. doi: 10.1159/000515835.

[31] Agarwal S, LeFevre AE, Lee J, et al. Guidelines for reporting of health interventions using mobile phones: mobile health (mHealth) evidence reporting and assessment (mERA) checklist. *Bmj.* 2016;352:i1174. 2016/03/19. doi: 10.1136/bmj.i1174.

[32] Rhon DI, Fritz JM, Kerns RD, et al. TIDieR-telehealth: precision in reporting of telehealth interventions used in clinical trials - unique considerations for the Template for the Intervention Description and Replication (TIDieR) checklist. *BMC Medical Research Methodology.* 2022;22(1):161. doi: 10.1186/s12874-022-01640-7.

[33] Dao KP, De Cocker K, Tong HL, et al. Smartphone-Delivered Ecological Momentary Interventions Based on Ecological Momentary Assessments to Promote Health Behaviors: Systematic Review and Adapted Checklist for Reporting Ecological Momentary Assessment and Intervention Studies. *JMIR Mhealth Uhealth.* 2021;9(11):e22890. 2021/11/23. doi: 10.2196/22890.

[34] Page MJ, McKenzie JE, Bossuyt PM, et al. The PRISMA 2020 statement: an updated guideline for reporting systematic reviews. *BMJ.* 2021;372:n71. doi: 10.1136/bmj.n71.

[35] Grainger R, Devan H, Sangelaji B, et al. Issues in reporting of systematic review methods in health app-focused reviews: A scoping review. *Health Informatics Journal.* 2020;26(4):2930-2945. doi: 10.1177/1460458220952917.

[36] Gasteiger N, Vercell A, Davies A, et al. Patient-facing genetic and genomic mobile apps in the UK: a systematic review of content, functionality, and quality. *Journal of Community Genetics.* 2022;13(2):171-182. doi: 10.1007/s12687-022-00579-y.

[37] Vercell A, Gasteiger N, Yorke J, et al. Patient-facing cancer mobile apps that enable patient reported outcome data to be collected: A systematic review of content, functionality, quality, and ability to integrate with electronic health records. *Int J Med Inform.* 2023;170:104931. 2022/12/04. doi: 10.1016/j.ijmedinf.2022.104931.

[38] Stoyanov S, Hides L, Kavanagh D, et al. Mobile app rating scale: a new tool for assessing the quality of health mobile apps. *JMIR mHealth uHealth.* 2015;3(1):e27. doi: 10.2196/mhealth.3422.

[39] Moher D, Schulz KF, Simera I, et al. Guidance for Developers of Health Research Reporting Guidelines. *PLOS Medicine.* 2010;7(2):e1000217. doi: 10.1371/journal.pmed.1000217.

[40] JBI Scoping Review Methodology Group. JBI manual for evidence synthesis: scoping reviews chapter. [Internet]. 2023. Available from: https://jbi.global/scoping-review-network/about

[41] Arksey H and O'Malley L. Scoping studies: Towards a methodological framework. *International Journal of Social Research Methodology* 2005;8(1):19-32. doi: 10.1080/1364557032000119616.

[42] Tricco AC, Lillie E, Zarin W, et al. PRISMA extension for scoping reviews (PRISMA-ScR): checklist and explanation. *Ann Intern Med.* 2018;169(7):467–473. doi: 10.7326/M18-0850.

[43] Moher D, Shamseer L, Clarke M, et al. Preferred reporting items for systematic review and meta-analysis protocols (PRISMA-P) 2015 statement. *Syst Rev.* 2015;4(1):1. 2015/01/03. doi: 10.1186/2046-4053-4-1.

[44] Clarke M, Oxman A, Paulsen E, et al. Appendix A: Guide to the contents of a Cochrane Methodology protocol and review. *Cochrane Handbook for Systematic Reviews of Interventions Version 510.* The Cochrane Collaboration, 2011.

[45] Levac D, Colquhoun H and O'Brien KK. Scoping studies: Advancing the methodology. *Implement Sci.* 2010;5(69):1-9. doi: 10.1186/1748-5908-5-69.

For peer review only

Appendix 1. PRISMA-P (Preferred Reporting Items for Systematic review and Meta-Analysis Protocols) 2015 checklist: recommended items to address in a systematic review protocol

Section and topic	Item No	Checklist item	Reported?
**ADMINISTRATIVE INFORMATION**			
Title:			
Identification	1a	Identify the report as a protocol of a systematic review	Yes (as scoping review protocol)
Update	1b	If the protocol is for an update of a previous systematic review, identify as such	N/A
Registration	2	If registered, provide the name of the registry (such as PROSPERO) and registration number	Yes
Authors:			
Contact	3a	Provide name, institutional affiliation, e-mail address of all protocol authors; provide physical mailing address of corresponding author	Yes
Contributions	3b	Describe contributions of protocol authors and identify the guarantor of the review	Yes
Amendments	4	If the protocol represents an amendment of a previously completed or published protocol, identify as such and list changes; otherwise, state plan for documenting important protocol amendments	N/A
Support:			
Sources	5a	Indicate sources of financial or other support for the review	Yes
Sponsor	5b	Provide name for the review funder and/or sponsor	Yes
Role of sponsor or funder	5c	Describe roles of funder(s), sponsor(s), and/or institution(s), if any, in developing the protocol	Yes
**INTRODUCTION**			
Rationale	6	Describe the rationale for the review in the context of what is already known	Yes
Objectives	7	Provide an explicit statement of the question(s) the review will address with reference to participants, interventions, comparators, and outcomes (PICO)	Yes (using SDMO acronym)
**METHODS**			
Eligibility criteria	8	Specify the study characteristics (such as PICO, study design, setting, time frame) and report characteristics (such as years considered, language, publication status) to be used as criteria for eligibility for the review	Yes
Information sources	9	Describe all intended information sources (such as electronic databases, contact with study authors, trial registers or other grey literature sources) with planned dates of coverage	Yes
Search strategy	10	Present draft of search strategy to be used for at least one electronic database, including planned limits, such that it could be repeated	Yes
Study records:			
Data management	11a	Describe the mechanism(s) that will be used to manage records and data throughout the review	Yes
Selection process	11b	State the process that will be used for selecting studies (such as two independent reviewers) through each phase of the review (that is, screening, eligibility and inclusion in meta-analysis)	Yes
Data collection process	11c	Describe planned method of extracting data from reports (such as piloting forms, done independently, in duplicate), any processes for obtaining and confirming data from investigators	Yes

For peer review only

Data items	12	List and define all variables for which data will be sought (such as PICO items, funding sources), any pre-planned data assumptions and simplifications	Yes
Outcomes and prioritization	13	List and define all outcomes for which data will be sought, including prioritization of main and additional outcomes, with rationale	Yes
Risk of bias in individual studies	14	Describe anticipated methods for assessing risk of bias of individual studies, including whether this will be done at the outcome or study level, or both; state how this information will be used in data synthesis	N/A
Data synthesis	15a	Describe criteria under which study data will be quantitatively synthesised	Yes
	15b	If data are appropriate for quantitative synthesis, describe planned summary measures, methods of handling data and methods of combining data from studies, including any planned exploration of consistency (such as I^2, Kendall's τ)	N/A
	15c	Describe any proposed additional analyses (such as sensitivity or subgroup analyses, meta-regression)	N/A
	15d	If quantitative synthesis is not appropriate, describe the type of summary planned	N/A
Meta-bias(es)	16	Specify any planned assessment of meta-bias(es) (such as publication bias across studies, selective reporting within studies)	N/A
Confidence in cumulative evidence	17	Describe how the strength of the body of evidence will be assessed (such as GRADE)	N/A

From: Shamseer L, Moher D, Clarke M, Ghersi D, Liberati A, Petticrew M, Shekelle P, Stewart L, PRISMA-P Group. Preferred reporting items for systematic review and meta-analysis protocols (PRISMA-P) 2015: elaboration and explanation. BMJ. 2015 Jan 2;349(jan02 1):g7647.

For peer review only

Appendix 2. Examples of the search strategy.

	SCOPUS	ACM Digital Library (The ACM Guide to Computing Literature)	APA PsycInfo (1806 to January Week 1 2024)	CINAHL Plus (EBSCO)	AMED (Allied and Complementary Medicine) 1985 to October 2023	Embase (1980 to 2024 Week 01)	Ovid MEDLINE(R) ALL (1946 to January 10, 2024)
**Technology**	mobile	mobile	Mobile Phones/	Mobile.mp	mobile.mp.	mobile phone/ or mobile.mp.	mobile.mp.
	smartphone	smartphone	Smartphones/	Smartphone/	Smartphone.mp	smartphone/	Smartphone/
	"cell phone"	"cell phone"	"cell phone".mp	“Cellular Phone”/	"Cell phone".mp	"cell phone".mp.	Cell Phone/
	mhealth	mhealth	mhealth.mp	Mhealth.mp	mhealth.mp.	mhealth.mp.	mhealth.mp.
	"mobile health"	"mobile health"	Mobile Health/	"mobile health".mp.	"mobile health".mp.	"mobile health".mp. or mobile health application/	"mobile health".mp.
	ehealth	ehealth	ehealth.mp.	eHealth.mp.	ehealth.mp.	ehealth.mp. or telehealth/	ehealth.mp.
	tele*	tele*	tele*.mp. Telemedicine/	Telehealth/	Telemedicine/ or tele*.mp.	tele*.mp. or telemedicine/	Telemedicine/ or tele*.mp.
**Review type**	((app OR apps OR application OR applications) W/5 review))	"app* review"	((App or apps or application or applications) adj5 review).mp.	(App or apps or application or applications) N5 review	((app or apps or application or applications) adj5 review).mp.	((app or apps or application or applications) adj5 review).mp.	((app or apps or application or applications) adj5 review).mp.
**Limits**	PUBYEAR > 2006	[E-Publication Date: (01/01/2007 TO 31/12/2024)]	yr="2007 -Current"	Publication Year: 2007-2024; Publication Date: 20070101-20241231	yr="2007 -Current"	yr="2007 -Current"	yr="2007 -Current"

For peer review only

Appendix 3. PRISMA (2020) checklist.

From: Page MJ, McKenzie JE, Bossuyt PM, Boutron I, Hoffmann TC, Mulrow CD, et al. The PRISMA 2020 statement: an updated guideline for reporting systematic reviews. BMJ 2021;372:n71. doi: 10.1136/bmj.n71

Section and Topic	Item #	Checklist item	Location where item is reported
**TITLE**			
Title	1	Identify the report as a systematic review.	
**ABSTRACT**			
Abstract	2	See the PRISMA 2020 for Abstracts checklist.	
**INTRODUCTION**			
Rationale	3	Describe the rationale for the review in the context of existing knowledge.	
Objectives	4	Provide an explicit statement of the objective(s) or question(s) the review addresses.	
**METHODS**			
Eligibility criteria	5	Specify the inclusion and exclusion criteria for the review and how studies were grouped for the syntheses.	
Information sources	6	Specify all databases, registers, websites, organisations, reference lists and other sources searched or consulted to identify studies. Specify the date when each source was last searched or consulted.	
Search strategy	7	Present the full search strategies for all databases, registers and websites, including any filters and limits used.	
Selection process	8	Specify the methods used to decide whether a study met the inclusion criteria of the review, including how many reviewers screened each record and each report retrieved, whether they worked independently, and if applicable, details of automation tools used in the process.	
Data collection process	9	Specify the methods used to collect data from reports, including how many reviewers collected data from each report, whether they worked independently, any processes for obtaining or confirming data from study investigators, and if applicable, details of automation tools used in the process.	
Data items	10a	List and define all outcomes for which data were sought. Specify whether all results that were compatible with each outcome domain in each study were sought (e.g. for all measures, time points, analyses), and if not, the methods used to decide which results to collect.	
	10b	List and define all other variables for which data were sought (e.g. participant and intervention characteristics, funding sources). Describe any assumptions made about any missing or unclear information.	
Study risk of bias assessment	11	Specify the methods used to assess risk of bias in the included studies, including details of the tool(s) used, how many reviewers assessed each study and whether they worked independently, and if applicable, details of automation tools used in the process.	
Effect measures	12	Specify for each outcome the effect measure(s) (e.g. risk ratio, mean difference) used in the synthesis or presentation of results.	

For peer review only - http://bmjopen.bmj.com/site/about/guidelines.xhtml

Section and Topic	Item #	Checklist item	Location where item is reported
Synthesis methods	13a	Describe the processes used to decide which studies were eligible for each synthesis (e.g. tabulating the study intervention characteristics and comparing against the planned groups for each synthesis (item #5)).	
	13b	Describe any methods required to prepare the data for presentation or synthesis, such as handling of missing summary statistics, or data conversions.	
	13c	Describe any methods used to tabulate or visually display results of individual studies and syntheses.	
	13d	Describe any methods used to synthesize results and provide a rationale for the choice(s). If meta-analysis was performed, describe the model(s), method(s) to identify the presence and extent of statistical heterogeneity, and software package(s) used.	
	13e	Describe any methods used to explore possible causes of heterogeneity among study results (e.g. subgroup analysis, meta-regression).	
	13f	Describe any sensitivity analyses conducted to assess robustness of the synthesized results.	
Reporting bias assessment	14	Describe any methods used to assess risk of bias due to missing results in a synthesis (arising from reporting biases).	
Certainty assessment	15	Describe any methods used to assess certainty (or confidence) in the body of evidence for an outcome.	
**RESULTS**			
Study selection	16a	Describe the results of the search and selection process, from the number of records identified in the search to the number of studies included in the review, ideally using a flow diagram.	
	16b	Cite studies that might appear to meet the inclusion criteria, but which were excluded, and explain why they were excluded.	
Study characteristics	17	Cite each included study and present its characteristics.	
Risk of bias in studies	18	Present assessments of risk of bias for each included study.	
Results of individual studies	19	For all outcomes, present, for each study: (a) summary statistics for each group (where appropriate) and (b) an effect estimate and its precision (e.g. confidence/credible interval), ideally using structured tables or plots.	
Results of syntheses	20a	For each synthesis, briefly summarise the characteristics and risk of bias among contributing studies.	
	20b	Present results of all statistical syntheses conducted. If meta-analysis was done, present for each the summary estimate and its precision (e.g. confidence/credible interval) and measures of statistical heterogeneity. If comparing groups, describe the direction of the effect.	
	20c	Present results of all investigations of possible causes of heterogeneity among study results.	
	20d	Present results of all sensitivity analyses conducted to assess the robustness of the synthesized results.	

For peer review only

Section and Topic	Item #	Checklist item	Location where item is reported
Reporting biases	21	Present assessments of risk of bias due to missing results (arising from reporting biases) for each synthesis assessed.	
Certainty of evidence	22	Present assessments of certainty (or confidence) in the body of evidence for each outcome assessed.	
**DISCUSSION**			
Discussion	23a	Provide a general interpretation of the results in the context of other evidence.	
	23b	Discuss any limitations of the evidence included in the review.	
	23c	Discuss any limitations of the review processes used.	
	23d	Discuss implications of the results for practice, policy, and future research.	
**OTHER INFORMATION**			
Registration and protocol	24a	Provide registration information for the review, including register name and registration number, or state that the review was not registered.	
	24b	Indicate where the review protocol can be accessed, or state that a protocol was not prepared.	
	24c	Describe and explain any amendments to information provided at registration or in the protocol.	
Support	25	Describe sources of financial or non-financial support for the review, and the role of the funders or sponsors in the review.	
Competing interests	26	Declare any competing interests of review authors.	
Availability of data, code and other materials	27	Report which of the following are publicly available and where they can be found: template data collection forms; data extracted from included studies; data used for all analyses; analytic code; any other materials used in the review.	

For peer review only

Appendix 4. PRISMA flowchart for new systematic reviews.

From: Page MJ, McKenzie JE, Bossuyt PM, Boutron I, Hoffmann TC, Mulrow CD, et al. The PRISMA 2020 statement: an updated guideline for reporting systematic reviews. BMJ 2021;372:n71. doi: 10.1136/bmj.n71

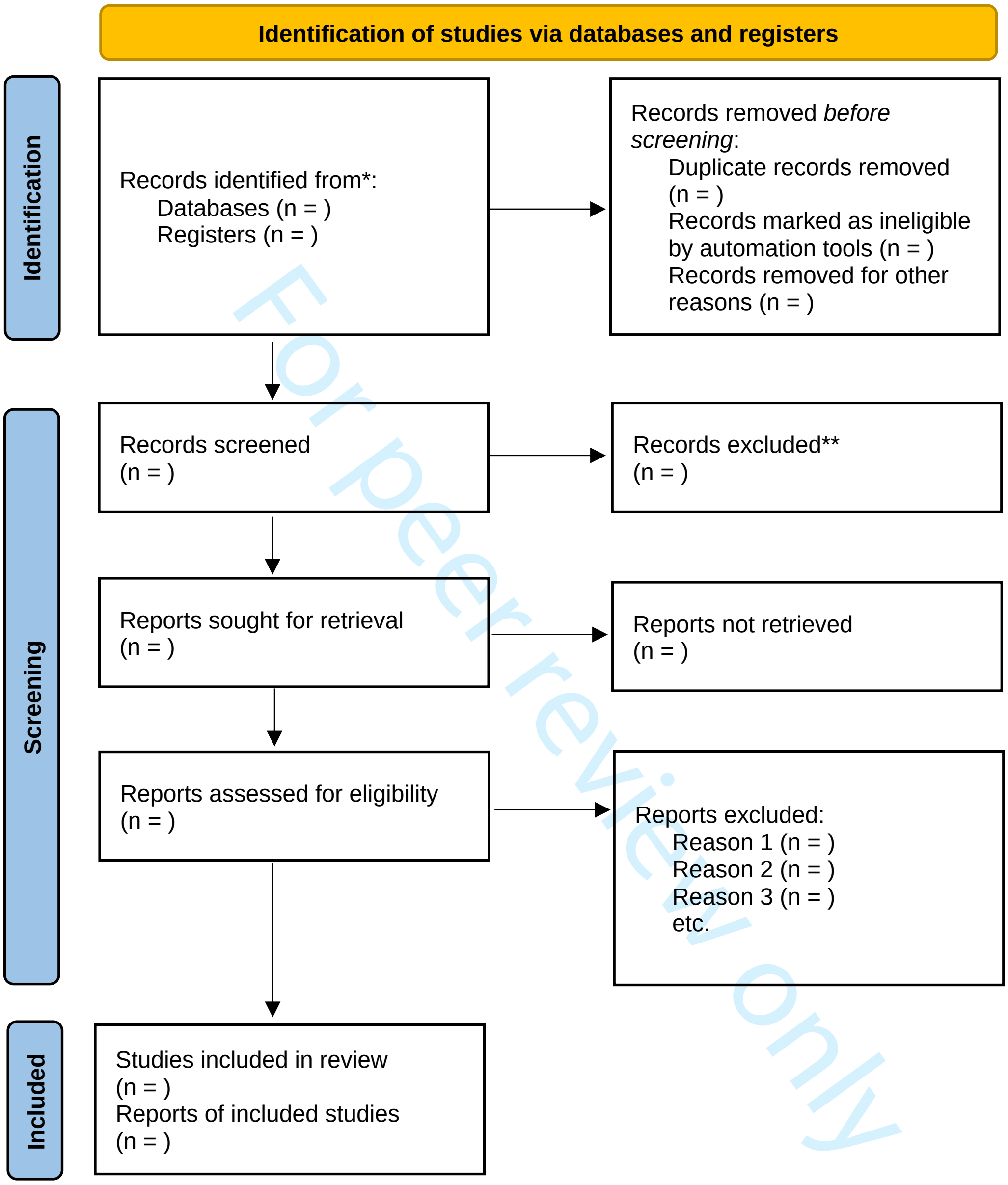

For peer review only

*Consider, if feasible to do so, reporting the number of records identified from each database or register searched (rather than the total number across all databases/registers).

**If automation tools were used, indicate how many records were excluded by a human and how many were excluded by automation tools.

For peer review only - http://bmjopen.bmj.com/site/about/guidelines.xhtml

Appendix 5. PRISMA flowchart for updated systematic reviews.

From: Page MJ, McKenzie JE, Bossuyt PM, Boutron I, Hoffmann TC, Mulrow CD, et al. The PRISMA 2020 statement: an updated guideline for reporting systematic reviews. BMJ 2021;372:n71. doi: 10.1136/bmj.n71

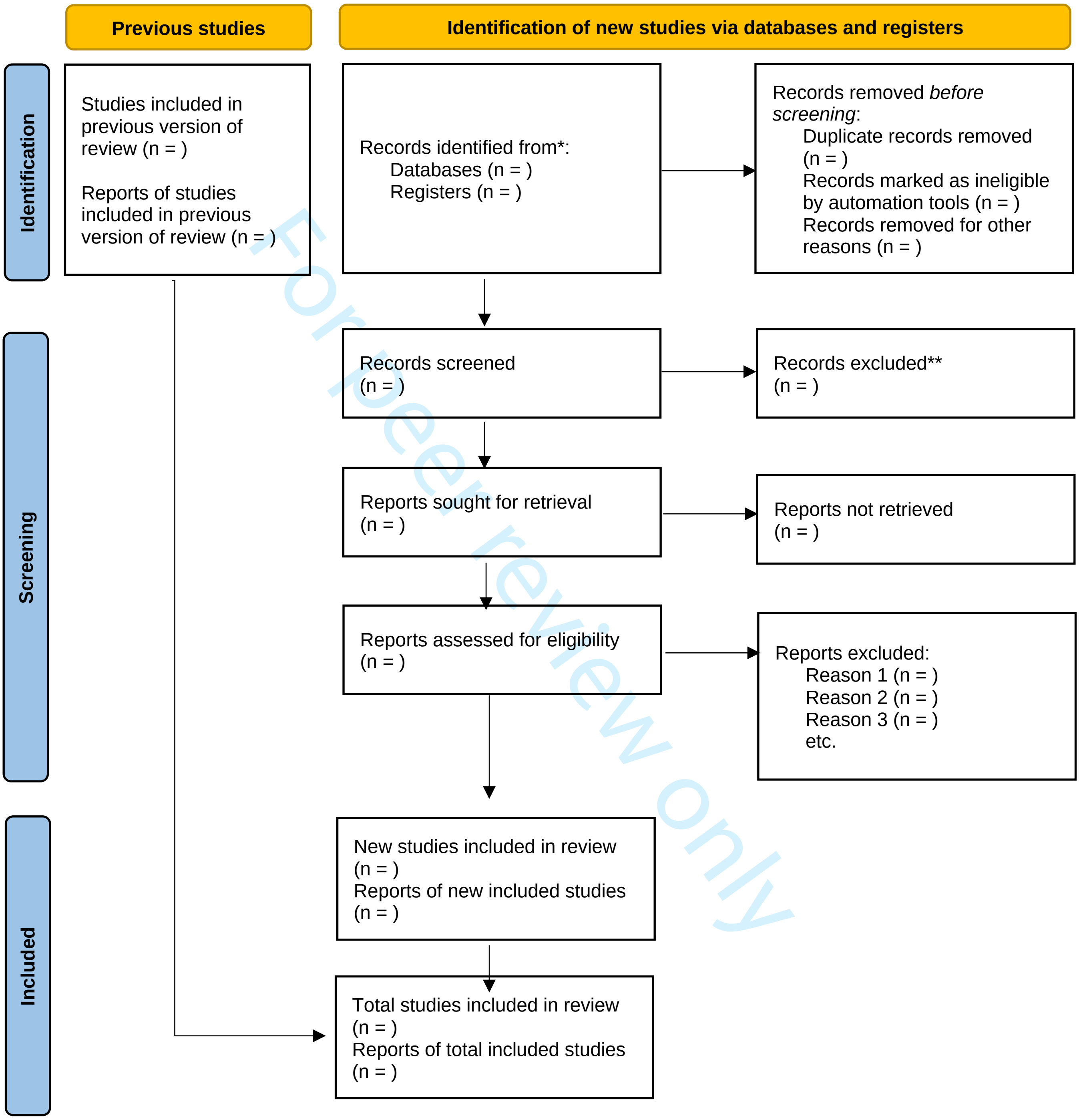

*Consider, if feasible to do so, reporting the number of records identified from each database or register searched (rather than the total number across all databases/registers).

**If automation tools were used, indicate how many records were excluded by a human and how many were excluded by automation tools.

For peer review only
